# Production of Wilson Disease Model Rabbits with Homology-Directed Precision Point Mutations in the *ATP7B* Gene Using the CRISPR/Cas9 System

**DOI:** 10.1038/s41598-018-19774-4

**Published:** 2018-01-22

**Authors:** Weihua Jiang, Lili Liu, Qiurong Chang, Fengying Xing, Zhengwen Ma, Zhenfu Fang, Jing Zhou, Li Fu, Huiyang Wang, Xingxu Huang, Xuejin Chen, Yao Li, Shangang Li

**Affiliations:** 1 Department of Laboratory Animal Science, School of Medicine, Shanghai Jiao Tong University, 200025 Shanghai, China; 2 School of Life Science and Technology, Shanghai Tech University, 201210 Shanghai, China

## Abstract

CRISPR/Cas9 has recently been developed as an efficient genome engineering tool. The rabbit is a suitable animal model for studies of metabolic diseases. In this study, we generated ATP7B site-directed point mutation rabbits to simulate a major mutation type in Asians (p. Arg778Leu) with Wilson disease (WD) by using the CRISPR/Cas9 system combined with single-strand DNA oligonucleotides (ssODNs). The efficiency of the precision point mutation was 52.94% when zygotes were injected 14 hours after HCG treatment and was significantly higher than that of zygotes injected 19 hours after HCG treatment (14.29%). The rabbits carrying the allele with mutant ATP7B died at approximately three months of age. Additionally, the copper content in the livers of rabbits at the onset of WD increased nine-fold, a level similar to the five-fold increase observed in humans with WD. Thus, the efficiency of precision point mutations increases when RNAs are injected into zygotes at earlier stages, and the *ATP7B* mutant rabbits are a potential model for human WD disease with applications in pathological analysis, clinical treatment and gene therapy research.

## Introduction

Genetically modified (GM) animals are powerful tools for studying gene functions and understanding the mechanisms of certain inherited human diseases. Various genetic alterations have been induced in mice since the 1980s through techniques such as fine mES cell culture^1,2^, chimera production^3^ and gene recombination techniques^4^ to produce unique animals. However, despite long-term attempts in many other species, mammalian germ line-transferred ESCs have been developed in only rats^5^. Another technique, somatic cell nuclear transfer (SCNT), was first used to clone sheep in 1997^6^, and this method of generating adult animals from a single cell has succeeded in creating genetically manipulated animals in many species, including sheep^7^, pigs^8,9^, ferrets^9^ and rabbits^10^. However, these methods depend on complicated techniques that can be performed by only well-equipped labs, and a substantial amount of time is required, particularly to produce conditional gene knock-out (CKO) or precision point mutation animals. In recent years, zinc finger nucleases (ZFNs), transcription activator-like effector nucleases (TALENs) and CRISPR/Cas9 gene editing systems have been developed as efficient genome engineering tools to generate gene knock-out (KO) animals via targeted nucleotide sequence cleavage and non-homologous end-joining (NHEJ) by microinjection into fertilized eggs^11–17^. Although new genetic manipulation techniques have potential disadvantages, they can provide a more convenient approach for generating CKO animals^18,19^ or repairing the mutated genome in adult animals via homologous recombination (HR) by large DNA fragments^20,21^.

Sequence variations named SNPs are important for increasing genetic variability but may also lead to hereditary diseases, such as Wilson disease (WD)^22^, Huntington disease^23^, cystic fibrosis^24^ or haemophilia^25^. In some hereditary diseases, gene functions are lost because of changes in amino acids. However, certain hereditary diseases are induced by enhanced or varied gene functions derived from SNPs^26,27^. These hereditary diseases cannot be simulated by KO animals. Therefore, precision point mutation animals produced by a special base knock-in (KI) are necessary. Rabbits are phylogenetically closer to primates than rodents, and they are physically large enough to permit non-lethal monitoring of physiological changes. Several transgenic or low-density lipoprotein receptor gene mutation rabbit models have been used for the study of lipoproteins and atherosclerosis because the lipid metabolism of rabbits is similar to that of humans^27–29^. Furthermore, several KO rabbits have been produced for different human hereditary diseases since new gene editing technologies were introduced^30–32^, and a reporter gene has been knocked into the ROSA26 locus in rabbits^33^. Given the extensive use of rabbits in research, the creation of precision point mutation rabbit lines is highly desirable.

In humans, ATP7B is an important protein that is mainly expressed in hepatocytes and contributes to transmembrane transport of copper. The dysfunction of ATP7B contributes to WD, an autosomal recessive genetic disorder of copper metabolism caused by a mutation in the *ATP7B* gene^34–36^. More than 600 mutations causing WD have been described. Different countries feature different hotspots. A missense mutation (p. Arg778Leu) in exon 8 of *ATP7B* is a major mutation affecting approximately 20% of people in certain parts of Asia^22,37–39^.

In this study, we used the CRISPR/Cas9 system to create a single amino acid substitute rabbit model for WD. The defined point mutations in the rabbit *ATP7B* gene were derived by microinjecting synthesized RNAs with ssODN donor sequences into zygotes, and the efficiency of homology-directed knock-in of point mutations was enhanced when RNAs were injected into younger zygotes.

## Methods

All experimental protocols, including animal care, the microinjection protocol and embryo transfer, met the approval guidelines (IACUC No. A-2015-002) established by the Ethics Committee of the School of Medicine of Shanghai Jiao Tong University.

### Plasmids

The pT7-cas9 vector and the gRNA cloning vector pCD-CAS were purchased from Biotechnologies Co., Ltd. (Nantong, China). The template sgF0 amplified from pCD-CAS by primers P-sgF0F and T7R1 (for primer sequences, see Supplementary Table S1) was used to produce different types of sgRNAs.

### sgRNA design

**Figure 1:**
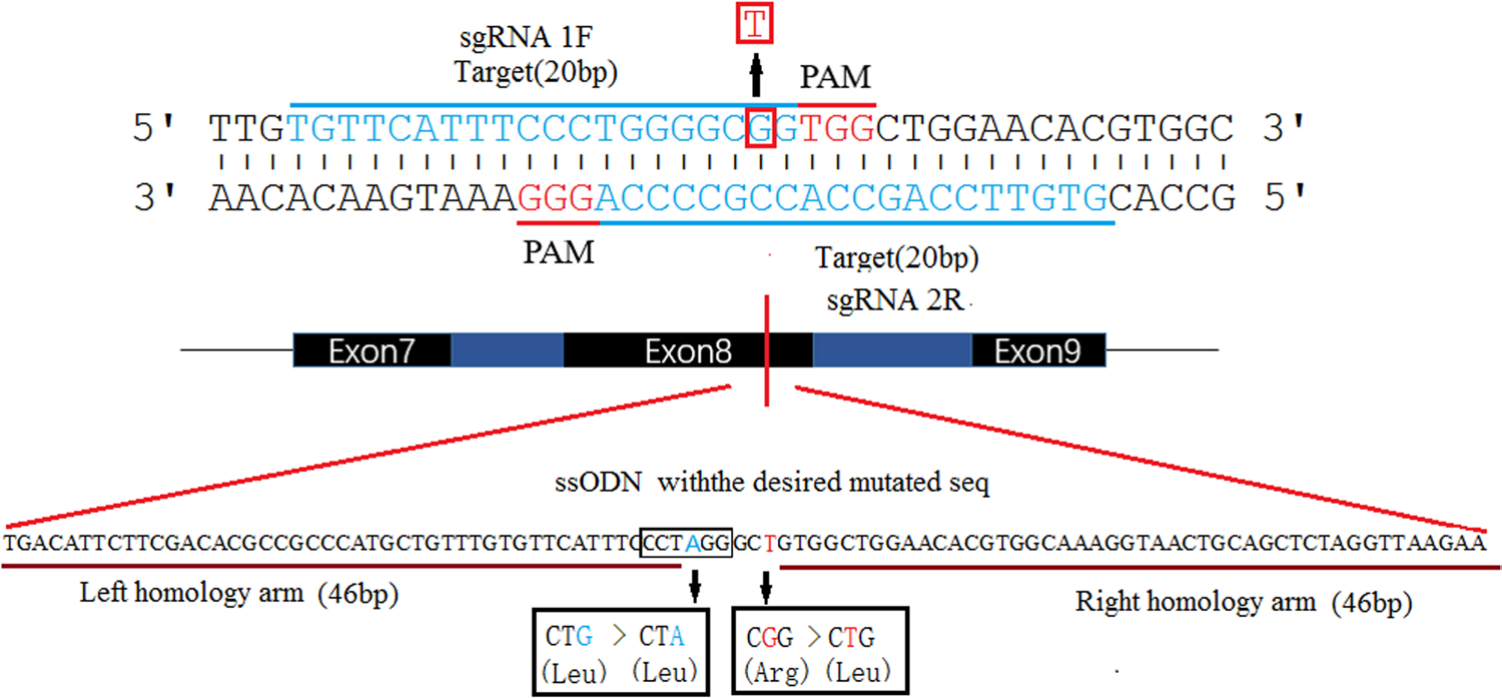
Diagram of two sgRNAs in rabbit *ATP7B* and the donor ssODNs. Two target sites of Cas9 in rabbit *ATP7B* in exon 8 (black bars represent the exons of the *ATP7B*; blue bars represent the introns). sgRNA1F and sgRNA2R are marked in blue, and the PAM sequences are presented in red. The base G in the cycle was replaced with T, thus resulting in Arg780Leu (CGG-CTG), producing a single precision point mutation. The *Bln* I digestion site marked in the box is introduced by a G-to-A (indicated in blue) replacement, which results in a synonymous mutation. The long sequence below is an ssODN with the desired mutations and homologous arms (left 46 bp and right 46 bp).

The results of protein BLAST showed that the motif consisting of the rabbit p. Arg780 is homologous to human p. Arg778. Two sgRNAs (sgRNA1F and sgRNA2R) were devised to target the sequence of exon 8 of rabbit ATP7B. The DNA sequences of sgRNA1F and sgRNA2R omitting NGG PAM were capped with a T7 promoter sequence, and they were named P-sg RNA1 and P- sg RNA2, respectively (Table S1). A missense mutation (p. Arg780Leu) was introduced by homologous recombination with single-stranded oligonucleotides (ssODNs). A *Bln* I digestion site was also introduced by a G-to-A (indicated in blue) replacement, which results in a synonymous mutation in the ssODN. The ssODNs with the desired mutation and two 46 bp homologous arms were synthesized by Thermo Fisher Scientific, Inc. (PAGE purified) (Fig. 1).

### RNA synthesis

The pT7-cas9 plasmid was digested with *Xba* I and purified by isopropanol precipitation. The linearized plasmid was transcribed into mRNA *in vitro* using a mMESSAGE mMACHINE T7 Ultra Transcription kit (Ambion, Texas, USA). Cas mRNA was purified by lithium chloride precipitation according to the manufacturer’s instructions. DNA templates of sgRNA1F and sgRNA1R were generated from sgF0 via PCR amplification by using the forward primers P-sgRNA1F and P-sgRNA1R with the reverse primer T7R1. The two abovementioned templates were reamplified with the T7 primer and T7R1. The products of the second PCR were purified and used for *in vitro* RNA synthesis with a MEGAshort T7 high yield Transcription Kit (Ambion, Texas, USA). The sgRNAs were purified with a MEGAclear-96 Purification of Transcription Reactions Kit in a 96-well format (Ambion, Texas, USA). Cas9 mRNA and sgRNAs were dissolved in TE buffer (Ambion, Texas, USA).

### Detection of external cutting efficiency of the sgRNAs

The efficacy of cleavage of sgRNAs *in vitro* was detected with a Guide-it sgRNA *in vitro* transcription and screening system kit (Takara, Dalian, China). The sgRNA-targeted fragments were amplified by PCR from wild rabbit DNA with the primers r78JDF and r78JDR. The cleavage reaction containing 20 ng of the experimental sgRNA sample and 100 ng of the sgRNA-targeted fragments was incubated at 37 °C for 1 hr and terminated by incubation at 70 °C for 10 min. The entire 10 μL reaction was analysed on a 2% TBE agarose gel.

### Microinjection

Mature female rabbits were superovulated by injection of 100 IU pregnant mare stimulation gonadotropin (PMSG, Ningbo A Second Hormone Factory, China) followed by 100 IU human chorionic gonadotropin (hCG, Ningbo A Second Hormone Factory, China) before they were mated. The fertilized eggs were flushed from the oviducts with pre-warmed HEPES-balanced RD medium (a 1:1 mixture of RPMI-1640 and DMEM supplemented with 10% foetal bovine serum (FBS, Gibco, USA), 2 mM/L HEPES, 2 mM/L L-glutamine and 100 μM/L NEAA), harvested in B2 medium prepared as previously reported^40^ and supplemented with 5% FBS before microinjection.

The mRNA and ssODN mixture included 20 ng/μL Cas9 mRNA and 10 ng/μL sgRNA1F and sgRNA2R. Fifty millimolar ssODNs in the final concentrations were prepared immediately before micromanipulation. Approximately 5 pL of the mixture was injected into the cytoplasm of each zygote by a 3 µm diameter pipette using a Piezo instrument (P mm 4 G, Prime Tech, Japan). After microinjection, zygotes were cultured in B2 medium in a humidified atmosphere containing 5% CO_2_ and 95% air at 38 °C for 24 hr.

### Embryo transfer

Cycles of female recipients were synchronized with zygote donors by injecting 100 IU hCG to induce ovulation. Approximately 20 embryos at the 4-cell or 8-cell stages were surgically transferred to both oviducts of each recipient through the infundibulum. Offspring were born naturally after 30 days of embryo transfer.

### Genotyping

Genomic DNA was extracted from ear punch tissues of newborn kits by using a TIANgen Genomic DNA kit (TIANGEN, Beijing, China). The fragments were amplified from genomic DNA by PCR using the primers r78JDF and r78JDR. First, the fragments were analysed on a 1.5% TBE agarose gel for detection of gross deletion, and then the PCR fragments were used for T7EN1 cleavage assay. Second, the PCR fragments were purified by isopropanol and digested with *Bln* I (Takara, Dalian, China) for analysis on a 2% TBE agarose gel. Furthermore, the PCR products were sequenced by Lifetech Co. (Shanghai, China).

### Off-target analysis

The potential off-target sites of the two sgRNAs were predicted using the online-based tool Cas9. The top three POTS were selected for each sgRNA according to the ranking scores. The potential off-target sites were analysed by BLAST searching of the predicted position in Oryctolagus cuniculus (rabbit) nucleotide sequences. Approximately 400–600 bp genomic fragments containing the off-target sites were amplified by PCR and sequenced. The off-target sequence and PCR primers used in this study are shown in Supplementary Table S2.

### Detection of ceruloplasmin in plasma

Rabbit ceruloplasmin (CP) concentrations in plasma were quantitatively detected between the knock-out or knock-in rabbits and wild rabbits. Blood was drawn from the ear vein, and the plasma was separated by centrifugation at 4 °C, 4000 rpm. Then, the operation was executed according to the instructions accompanying a Rabbit CP ELISA Kit (Mlbio, Shanghai, China) on Spectramax i3 (Molecular Devices, USA).

### Detection of copper in plasma and liver function indicators

Approximately 400 μL of plasma was treated by using a Copper Assay Kit according to the manufacturer’s instructions (Jiancheng Bioengineering Institute, Nanjing, China) and was analysed at a visible light wavelength of 580 nm with a biochemical analyser (Thermo Scientific, USA). Liver function indicators, including albumin (ALB), total protein (TP), alkaline phosphatase (ALP), glutamyl transferase (GGT), alanine aminotransferase (ALT) and aspartate aminotransferase (AST), were detected with the same machine using kits supplied by Ikon Biotechnology Co., Ltd. (Zhejiang, China).

### Quantitative detection of tissue copper concentration

*ATP7B* was mainly expressed in the liver and kidney. Therefore, 0.5 g of liver and kidney was extracted from the homologous mutation rabbits and the wild-type rabbits after euthanasia. All these reagents and protocols were implemented according to the instructions accompanying the Tissue Copper Colori Assay Kit (GENMED, Shanghai, China). The tissues were cleaned and placed in a cryopreservation tube in liquid nitrogen overnight. The next day, the tissues were homogenized to obtain a supernatant for the detection of copper ion concentration at 580 nm using a spectrophotometer (Eppendorf, German).

### HE staining of hepatic tissue

The liver tissues of the rabbits were treated with 10% formalin for 24 hr, dehydrated in 75% ethanol for 24 hr followed by dehydration in different grades of alcohol, and vitrified with dimethylbenzene. After the tissues were embedded in paraffin, 5 μm longitudinal sections were stained with haematoxylin solution for 5 min and washed with running water and then stained with eosin solution for 20 sec. Finally, xylene was applied for clearing, and the samples were observed under a light microscope (Nikon, Japan) after the samples were sealed with neutral balsam on slides.

### Data presentation and statistical analyses

For all parameters, the parameters were test for three times, the average of three number and the error were show in the data, and expressed as means ± standard error. The mutation kits obtained by microinjection at different times were examined by a chi-square test to compare the KI frequencies obtained when zygotes were injected 19 hr after HCG treatment with the KI frequencies obtained when zygotes were injected 14 hr after HCG treatment. The significant differences in the biochemical index between muted and WT rabbits were analysed using Student’s *t*-test. The level of statistical significance was set to P < 0.05.

## Results

### Production of sgRNA and CRISPR/Cas9 RNA

Cas9 mRNA was purified after *Xba* I digestion and transcription. The SgF0 fragment was amplified from the pCD-CAS plasmid (Biomics Biotech, China) by PCR with primers and used as a template to amplify different target DNA fragments for transcription. After transcription and purification, two sgRNAs were obtained with sufficient concentration and quality according to biometric spectrophotometry.

### Detection of the *in vitro* cutting efficiency of sgRNAs

**Figure 2:**
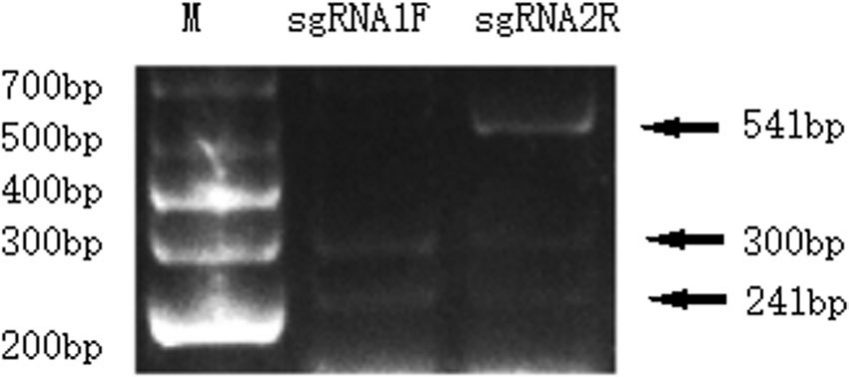
Detection of the efficiency of sgRNAs cutting *in vitro*. The full length of the fragment is 541 bp. The two fragments that were found after digestion measure 241 bp and 300 bp. M: DL1000 DNA Marker.

The target fragments (541 bp) were cut by the Cas9 nuclease and combined with two sgRNAs. The Cas9 nuclease and sgRNA1F digested the fragment into two bands with lengths of nearly 240 bp and 300 bp, suggesting that the fragment was fully cut and that the efficiency was 100%. Three bands at 541 bp, 300 bp and 241 bp were detected after sgRNA2R digestion (Fig. 2). The results demonstrated that sgRNA1F had a higher cutting efficiency than sgRNA2R.

### Generation of *ATP7B* precision point mutation (KI)/KO rabbits by using the CRISPR/Cas9 system through zygote injection

To generate an amino acid substituted rabbit model via the CRISPR system, a mixture of 20 ng/μL Cas9 RNA, 10 ng/μL sgRNA1F, 10 ng/μL sgRNA2R and 50 mM ssODNs was co-microinjected into rabbit pronuclear stage embryos obtained from the donor rabbits 19 hours after HCG treatment. The injected embryos were cultured overnight in B2 medium. Sixty-two embryos at the 4-cell or 8-cell stage were transferred into three recipients. Fourteen pups were produced, including 2 (14.29%) pups with precision point mutations in the Arg780Leu locus and 7 (50%) KO pups. To improve the efficiency of point mutations, 56 fertilized eggs obtained at 14 hrs after HCG were injected with the mixture of RNAs and ssODNs. Consequently, 17 pups were obtained, of which 9 (52.94%) carried a point mutation and 5 (29.41%) were KO pups (Table 1).

**Table 1:** The results of mutation pups obtained by microinjection at different times.

	Zygotes microinjected	pups obtained (%transferred)	Pups with KO (% pups obtained)	pups with KI (% pups obtained)
Zygotes injected 19 hr after HCG treatment	62	14 (22.58%)	7 (50%)	2 (14.29%)^a^
Zygotes injected 14 hr after HCG treatment	56	17 (30.36%)	5 (29.41%)	9 (52.94%)^b^

KO: knock-out, KI: knock-in (point mutation).

Chi-square analysis demonstrated significant differences between a and b (*P < 0.05).

**Figure 3:**
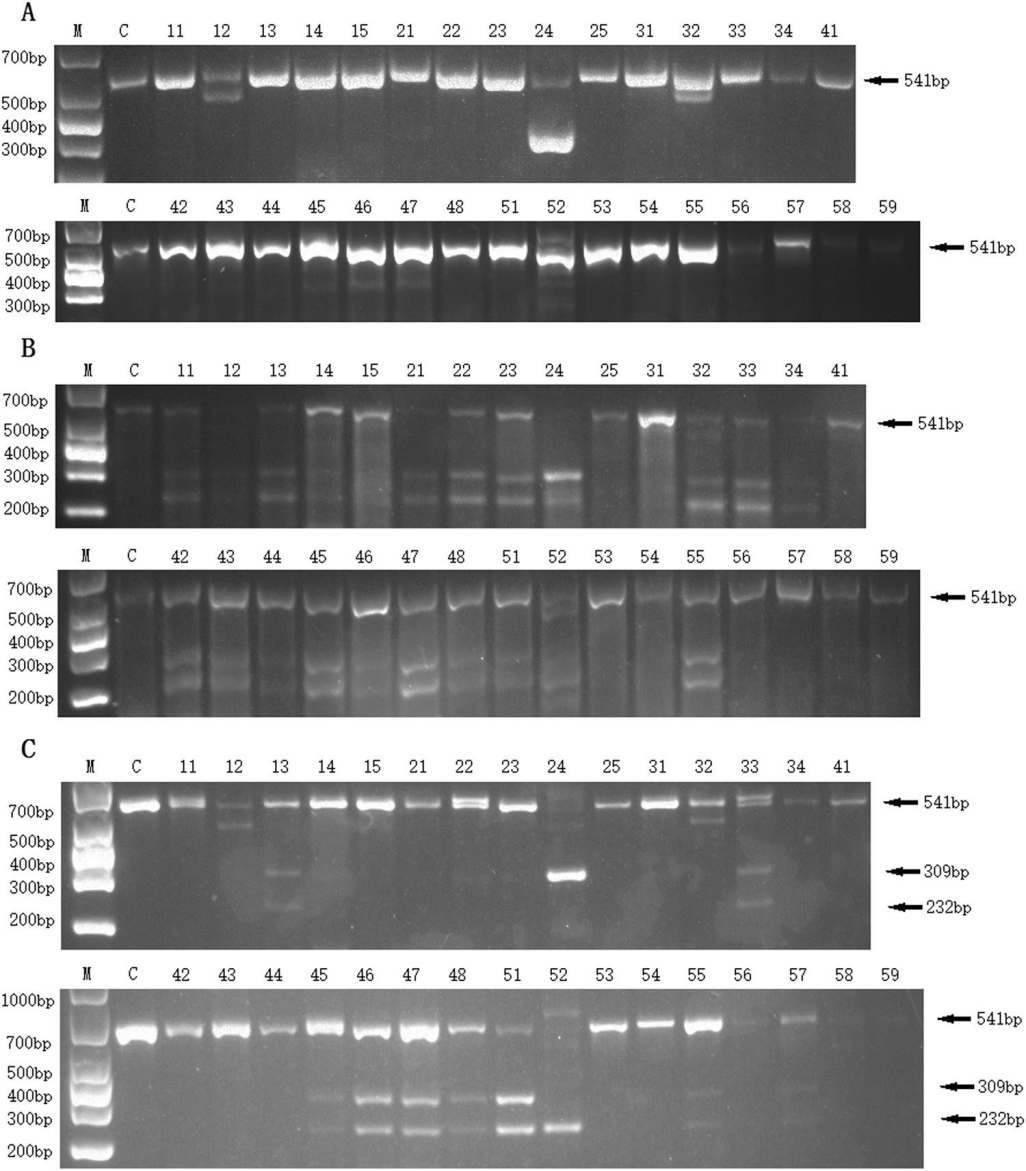
Genotype analysis of the 32 pups. (**A**) PCR analysis of the genomic DNA from the 32 pups. (**B**) T7EN1 cleavage assay of the 32 pups. The letter C represents wild-type rabbits. (**C**) The *Bln* I digestion assay of the 32 pups. The letter C represents wild-type rabbits. The numbers from 11 to 59 represent the F0 pups. M: DL1000 DNA Marker (Takara, Dalian, China).

**Figure 4:**
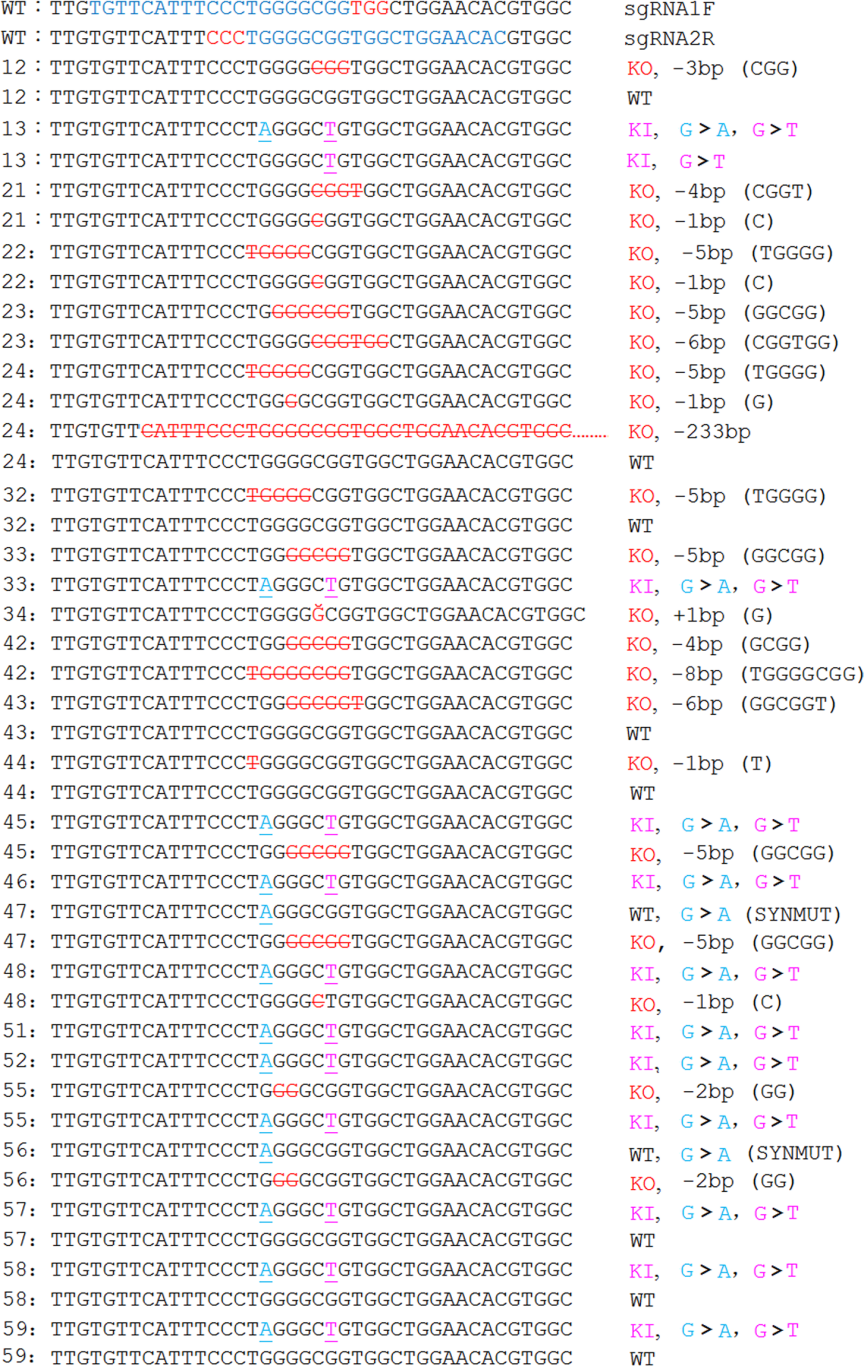
Genotypes of the mutant pups. The target site of sgRNA1F is highlighted in red, and the PAM sequence is shown in green. The target site of sgRNA2R is shown inside the box, and the PAM sequence is labelled by a red wavy line. The point mutation is underlined in magenta, and the *Bln* I endonuclease cutting site is underlined in blue. The mutated base numbers and their sequences are shown on the right (+, insertion; −, deletion). A > G indicates that G was substituted by A, and T > G indicates that G was substituted by T. WT, wild type; KO, knock-out; KI, knock-in; SYNMUT, synonymous mutation.

The genomic DNA from each pup was amplified by PCR, and TBE agarose analysis showed whether gross deletions were made in the target region. The PCR data showed that rabbits 12, 24, 32, 45, 46, 47 and 52 had short bands (Fig. 3A), indicating that distinct deletions had occurred; for example, rabbit 24 had a 233 bp deletion (Fig. 4). The T7EN1 cleavage assay, which can detect the mismatch in the centre of a sequence derived either from an indel and/or the KI, showed that samples from rabbits 11, 12, 13, 14, 21, 22, 23, 24, 32, 33, 34, 42, 43, 44, 45, 46, 47, 48, 51, 52 and 55 were cut into two bands (Fig. 3B). Finally, the PCR products were digested by the *Bln* I for detection of the KI. The *Bln* I digestion assay showed that samples from rabbits 13, 33, 45, 46, 47, 48, 51, 55 and 57 were cleaved into two bands (232 bp and 309 bp) (Fig. 3C). The results of Sanger sequencing showed that there were eleven point mutation and twelve KO pups in total. All genotypes of mutant rabbits are displayed in Fig. 4; five of them are double KO (rabbit 21, 22, 23, 34 & 42), four of them are KO/KI (rabbit 33, 45, 48 & 55), four of them are double KI (rabbit 13, 46, 51, & 52), and one of them has three types of KO and WT (rabbit 24). The ssODNs used for recombination had two bases changed (G > A & G > T); however, three rabbits carried one base replacement [G > T(rabbit 13) or G > A(rabbit 47 & 56)].

### Heritability of the *ATP7B* point mutation and KO pups

**Figure 5:**
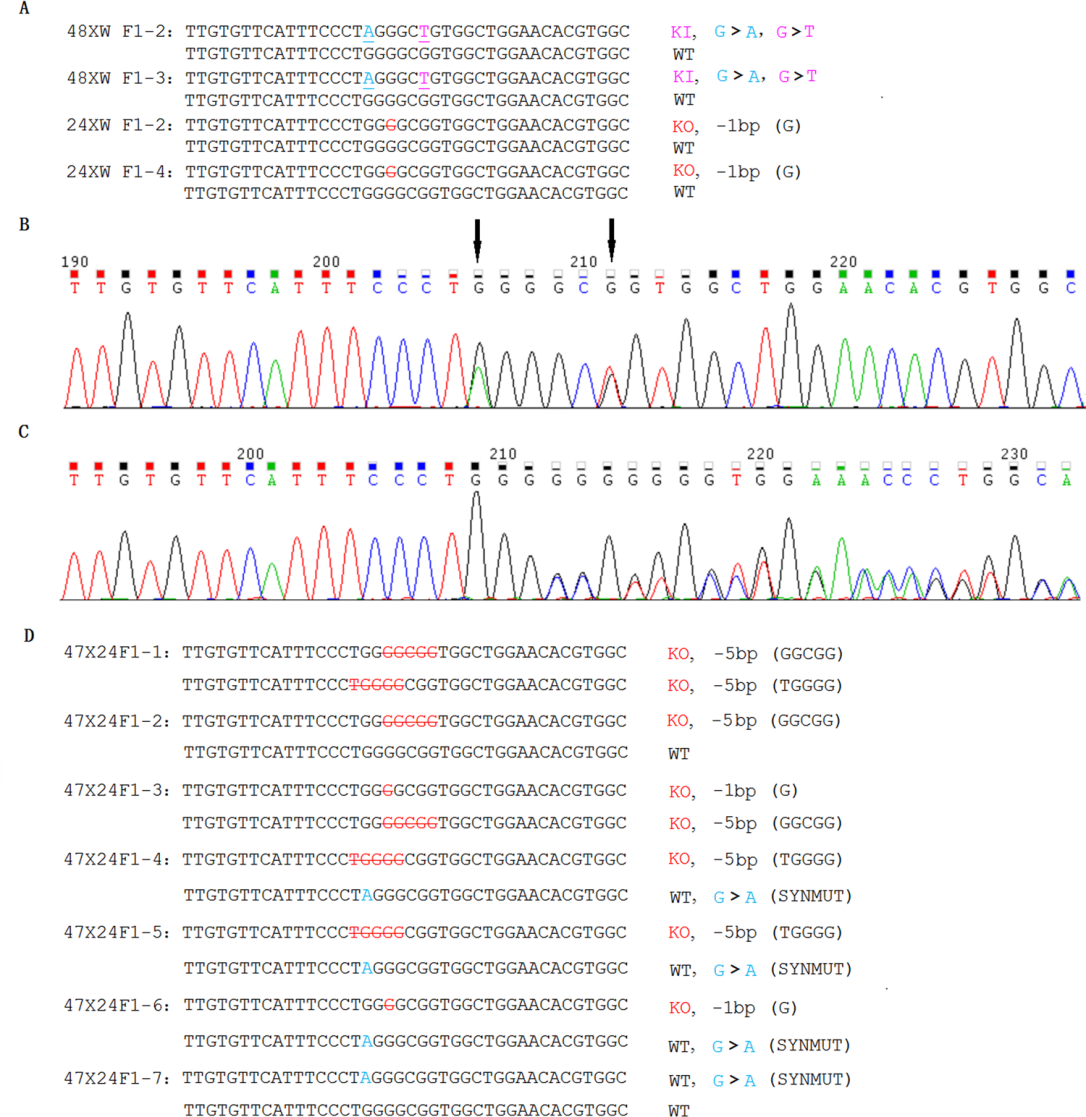
Results of genotype and sequencing chromatograms of F1 pups from different parents. (**A**) The analysis of sequences of F1 pups obtained from mating wild rabbits with F0–48 and F0–24. The point mutation is underlined in magenta, and the endonuclease cutting site *Bln* I is underlined in blue. The mutated base numbers and their sequences are shown on the right (−, deletion). A > G indicates that G was substituted by A, and T > G indicates that G was substituted by T. WT, wild type; KO, knock-out; KI, knock-in (point mutation). (**B**) Sequencing chromatogram of F1 obtained from F0–48 mated with a wild-type rabbit. The black arrows represent the sites of synonymous mutations and point mutations. (**C**) Sequencing chromatogram of an F1 pup obtained from F0–24 mated with wild-type rabbits. (**D**) The analysis of sequences of F1 obtained from F0–47 mated with F0–24. The endonuclease cutting site *Bln* I is underlined in blue. The mutated base numbers and their sequences are shown on the right (−, deletion). A > G indicates that G was substituted by A. WT, wild type; KO, knock-out; SYNMUT, synonymous mutation.

To determine whether the genotype of the *ATP7B* precision point mutations and KOs could be stably transmitted to offspring, adult mutant rabbits were used for reproduction. When the male rabbit F0–48 was mated with a wild-type (WT) female rabbit, two rabbits carrying the point mutation were derived from three offspring. When the male rabbit F0–24 was mated with the WT female rabbits, two rabbits carrying the KO were derived from four offspring (Fig. 5A–C).

To produce compound heterozygote offspring, the female rabbit F0–47 was mated with F0–24, and 7 offspring were born. In the 7 offspring, two pups were compound heterozygotes, three pups carried KO and a synonymous mutation genotype, one pup carried KO and a wild genotype, and one pup carried a synonymous mutation and wild genotype (Fig. 5D).

### Off-target detection

Off-target effects are a crucial concern in using the CRISPR/Cas9 system. To detect the occurrence of off-target mutations in all 23 newborn genetically modified rabbits, the three highest-scoring sequences of potential off-target sites for each sgRNA were predicted by the CRISPR design online tool (Table S2). After PCR amplification and Sanger sequencing, 60.87% of the mutant rabbits were found to be off-target at the highest-scoring-potential off-target sites of sgRNA1F (Fig. S6). Moreover, 8.69% of the wild-type rabbits were off-target at the same site. However, there were no off-target sequences at the other score sites of the sgRNAs (Figs S7 and S8).

In addition, fourteen F1 offspring were tested for germline transmission of the off-target mutations, five (35.71%) of which carried the off-target genotype. The rabbits that carried the off-target genotype were two of three rabbits in the offspring produced by mating F0–48 with a WT female rabbit. The genotype was also detected in three of seven rabbits in the offspring produced by F0–47 mated with F0–24. Eleven of these fourteen F1 offspring carried mutations, and six of them did not carry the off-target genotype.

### The ceruloplasmin level in *ATP7B* mutant rabbits

**Figure 6:**
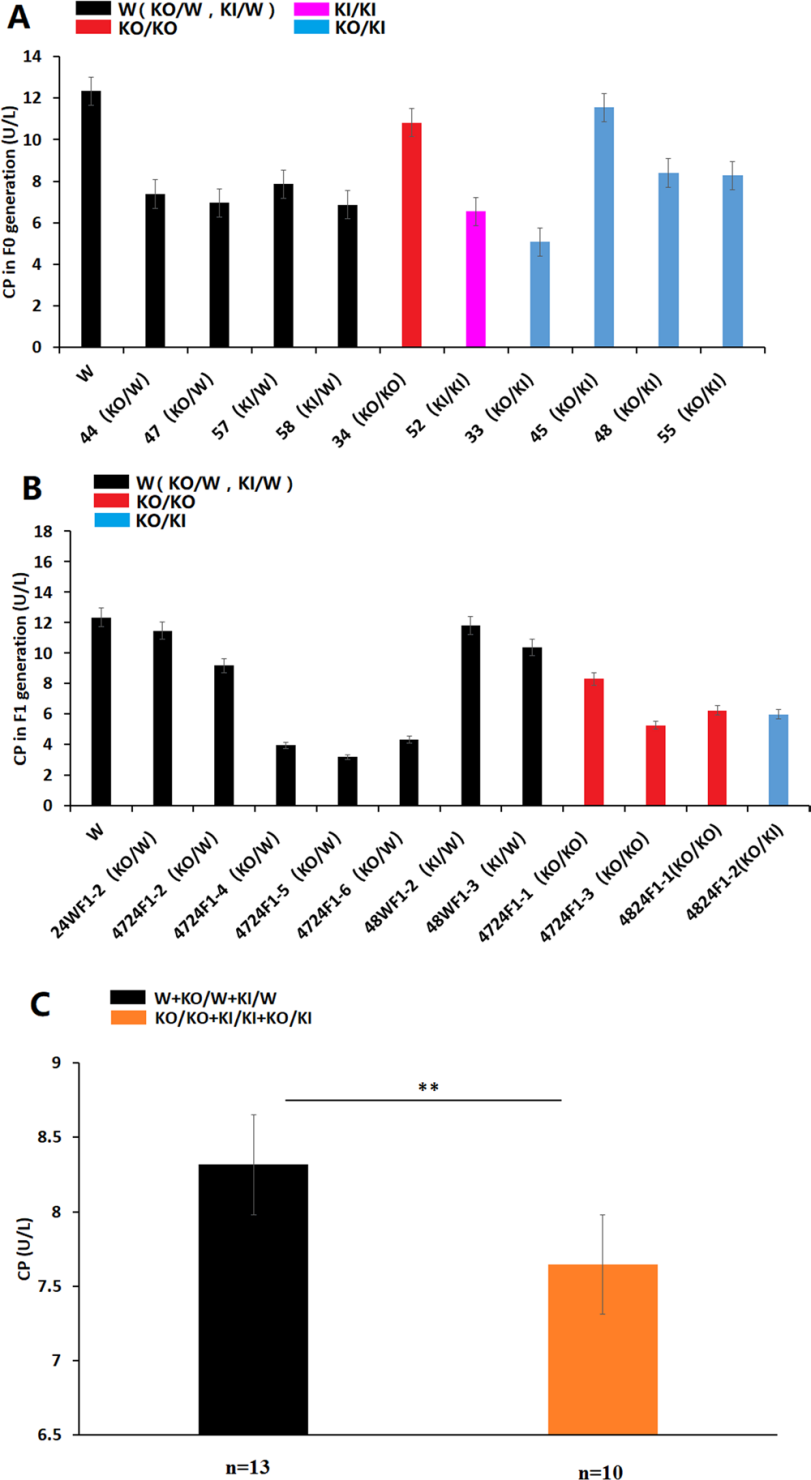
Levels of ceruloplasmin in different genotype rabbits. (**A**) The ceruloplasmin level in the *ATP7B* mutant rabbits of F0. (**B**) The ceruloplasmin level in the *ATP7B* mutant rabbits of F1. (**C**) Differences in the ceruloplasmin concentration in the homozygous and heterozygous mutations in F0 and F1. There was a significant difference between the homozygous mutations and wild-type individuals (*P < 0.05). W: wild type, KO: knock-out, KI: knock-in (point mutation).

Ceruloplasmin is the major carrier of copper in the blood and is typically low in patients with WD. The ceruloplasmin level showed a downward trend in the mutant rabbits of the F0 (Fig. 6A) and F1 (Fig. 6B) generation compared with that observed in the WT rabbits. In all the above 23 samples of rabbit of F0 and F1 (Fig. 6A,B), there was a significant difference between the homozygous (KO/KO, KO/KI & KI/KI) and WT individuals (W/W, W/KO & W/KI) (*P < 0.05) (Fig. 6C).

### Plasma copper in the *ATP7B* mutant rabbits

**Figure 7:**
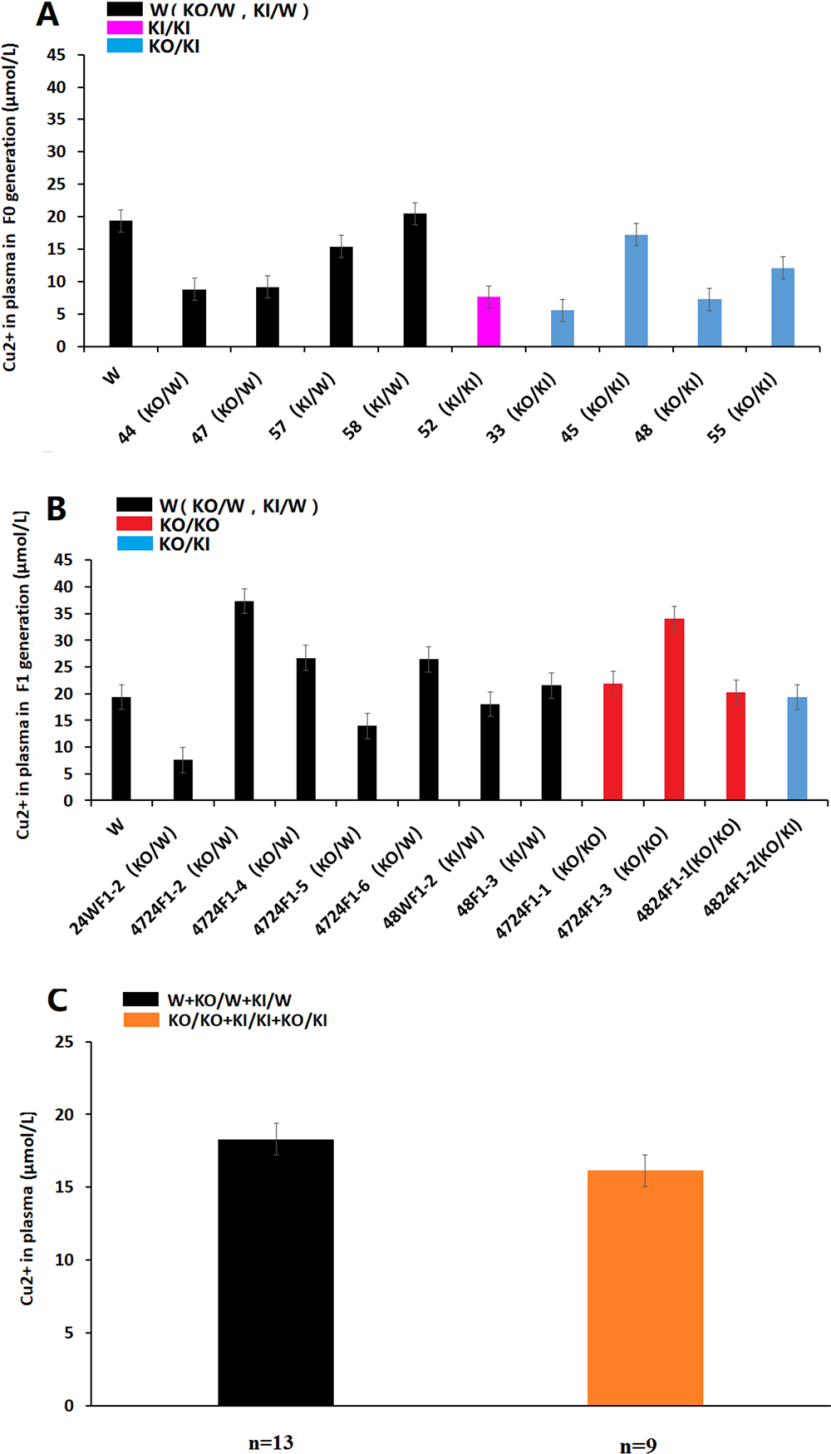
Levels of copper in plasma in rabbits with different genotypes. (**A**) The plasma copper level in the *ATP7B* mutant rabbits of F0. (**B**) The plasma copper level in the *ATP7B* mutant rabbits of F1. (**C**) Differences in the plasma copper level for homozygous and heterozygous mutations. There was no significant difference between the homozygous mutations and wild-type individuals (P > 0.05). W: wild type, KO: knock-out, KI: knock-in (point mutation).

The plasma copper in patients with WD is usually decreased in proportion to the decreased ceruloplasmin in the circulation, whereas in patients with severe liver injury, plasma copper may be within the normal range despite the lower ceruloplasmin level. The results of plasma copper detection showed that most mutant rabbits had a lower plasma copper level than that of the WT rabbits in generation F0 (Fig. 7A). However, a minority of the rabbits showed a higher level in generation F1 than that of the WT rabbits (Fig. 7B). In all the above22 samples of rabbit of F0 and F1 (Fig. 7A,B). There was no significant difference (P > 0.05) between the homozygous and WT individuals (Fig. 7C).

### The liver function indicators in plasma in the *ATP7B* mutant rabbits

Hepatocyte damage is one of the main features of WD. The following results were obtained when liver function indicators were measured in the F0 and F1 rabbits, and the above data were also compared between homozygous and WT individuals (Fig. S1). The levels of ALB and TP in the homozygous plasma were lower than those in the WT individuals, whereas the levels of ALB, ALP, GGT, ALT and AST all increased to some extent in all F0 and F1 rabbits (Fig. S1). The differences between the homozygous and WT individuals in ALB, ALP, GGT and ALT were significant (*P < 0.05), whereas the difference between the heterozygous and mutation-type individuals in TP and AST was not significant (P > 0.05).

### Tissue copper in the *ATP7B* mutant rabbit

**Figure 8:**
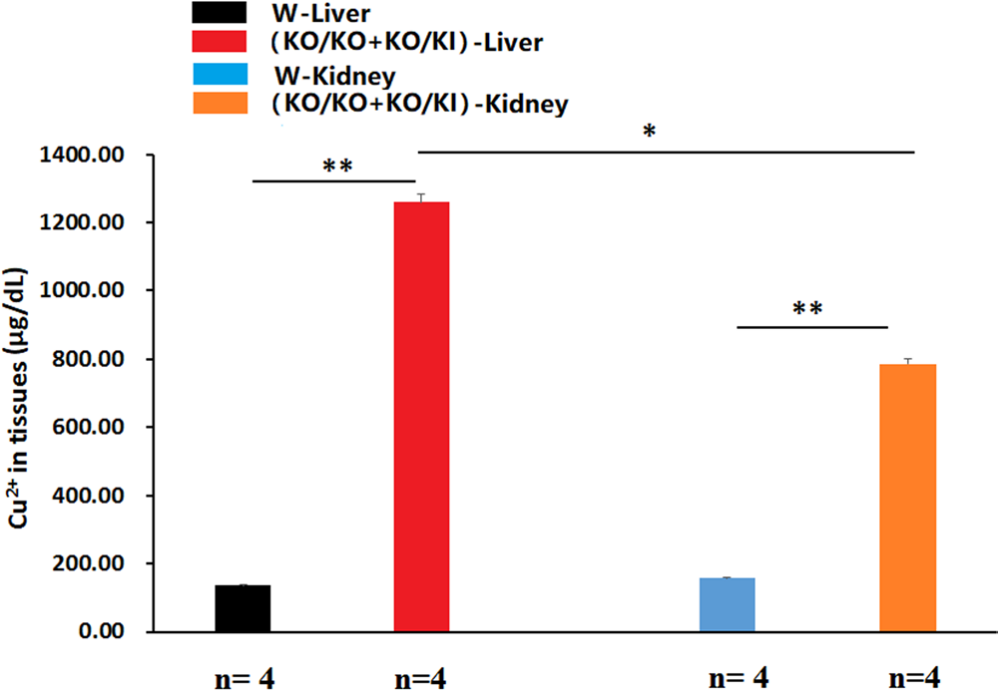
Amount of tissue copper in the mutant rabbits. W: wild type, KO: knock-out, KI: knock-in (point mutation) (**P < 0.01, *P < 0.05).

The disorder of copper metabolism in WD consistently results in pathological accumulation of copper in many organs and tissues, particularly in the liver and kidney. Liver and kidney tissue of four homologous rabbits ((F0 KI/KO & KI/KI, F1 KO/KO & KO/KI) and four WT litter-mate rabbit were detected for the accumulation of copper. The outcomes of tissue copper detection showed that the copper in the livers of the *ATP7B* mutant rabbits was 9 times higher than that in the wild rabbits (**P < 0.01). The copper in the kidney was 5 times higher than that in the wild rabbits (**P < 0. 01). In addition, the liver copper was higher than that in the kidneys of mutant rabbits (*P < 0.05) (Fig. 8).

### Morphological observation of the mutant liver

**Figure 9:**
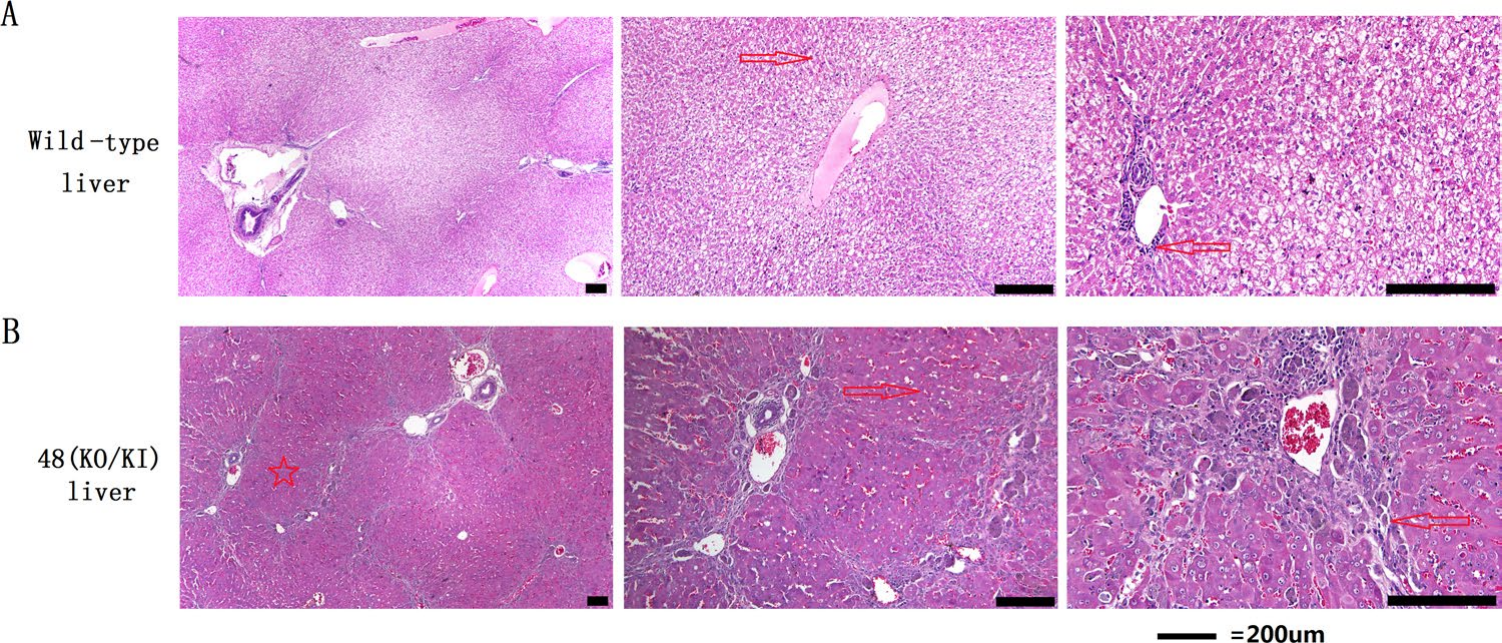
HE staining of livers. (**A**) HE staining of a wild-type rabbit liver. (**B**) HE staining of a KO/KI mutation-type liver (rabbit 48). Scale bars = 200 μm. Hepatic pseudo-lobule (☆), connective tissue (←) and hepatic cell cords clear (→) are marked by different symbols.

HE staining demonstrated that liver of the mutant rabbits exhibited morphological changes relative to those of the wild-type rabbits. There was clear connective tissue proliferation in the livers of the mutant rabbits, and a hepatic pseudo-lobule appeared. Additionally, hepatic cell cords were not distinct (Fig. 9).

## Discussion

This research was based on the use of micro-manipulation technology combined with an efficient and specific CRISPR/Cas9 system to make the *ATP7B* gene point mutation occur at the high homology site of the 780th amino acid arginine through homologous recombination. This disease model simulates the major mutation type in Asians with WD.

WD is a worldwide disease caused by a mutation in the *ATP7B* gene. The mutation of *ATP7B* may cause ceruloplasmin synthesis disorder and biliary copper excretion disorder, thus resulting in excessive copper deposition in various organs, particularly the liver and kidneys. The hotspot mutation in Europeans is in exon 14, whereas Arg778Leu in exon 8 in Chinese and Koreans is the first hotspot^38,41,42^. Protein-protein BLAST analysis indicated that the homology of the amino acid sequences between the human and the rat or mouse ATP7B proteins is only 50% (Fig. S2,S3). However, the homology is as high as 87% between human and rabbit ATP7B proteins (Fig. S4). Moreover, the homology of exon 8 between humans and rabbits is up to 94.87%, and the 778^th^ arginine in humans is homologous to the 780^th^ arginine in rabbits (Fig. S5). Consequently, we chose exon 8 of the rabbit as the point mutation site and designed a pair of sgRNAs combined with ssODNs to produce mutations.

CRISPR/Cas9 has been developed as an efficient genome engineering tool to introduce a site-directed point mutation with ssODNs^43,44^. In the process of producing the point mutation, we found that the time interval between HCG treatment and RNA injection was related to the efficiency of point mutation. The efficiency of point mutation rabbits (52.94%) 14 hours after injection of HCG was significantly higher than that 19 hours after injection of HCG (14.29%)(*p < 0.05) (Table 1). One possible reason for this result is that the nuclear membrane was not completely formed at the earlier time, thus facilitating entry of the sgRNA-Cas9 complex and ssODNs into the nucleus. Another possible reason is that there is a shorter length of time from fertilization to the first cleavage in rabbit embryos than in mouse embryos. Early injection may provide sufficient time for Cas9RNA translation and prolong the exposure to the sgRNACas9 complex and ssODNs before the first cleavage. The results suggested that adjustment of the interval between the HCG processing time and RNA injection time may affect the efficiency of precision point mutations in similar studies.

In this study, two sgRNA were devised to cut the target sequence. The *in vitro* cutting assay showed that sgRNA1F had a higher cutting efficiency than sgRNA2R; however, sgRNA2R has a lower off-target rate, as predicted online. Therefore, the two sgRNAs were used for all experiments. The results of DNA sequencing showed that most indel mutations occurred between the two PAMs of the couple sgRNAs. These two sgRNAs cutting may also facilitate precision point mutations for HDR repair using Cas9/gRNAs, and ssODNs utilize synthesis-dependent strand annealing pathways when double-strand lesions are derived by Cas9^45^. When the ssODNs were annealed with the overhang broken strand end, the complementary strands were synthesized followed by flap cleavage, ligation and single-strand gap repair with another broken strand end. The sequence of another broken strand end may disturb the sequence of the ssODNs, and thus, only one part of the point mutation was introduced, e.g., only one base was replaced in three rabbits (Fig. 4).

Eight heterozygous rabbits grew into adults, and three of them were mated with each other or with wild-type rabbits. Genotype analysis of the F1 generation showed that the genotypes of the *ATP7B* point mutation and KO were stably transmitted to the offspring. Additionally, complex heterozygous and heterozygous rabbits were also produced from the mutant parents.

To address concerns about the possibility of the CRISPR/Cas9 system generating “off-target” changes to the rabbit genome, we analysed the genomes of all the founder rabbits by using a combination of predictive bioinformatics to identify possible off-target sites, PCR to allow for amplification and Sanger sequencing to identify off-target effects in these sites. The sgRNA1F produced off-target mutations—up to 60.87% of the highest-scoring-potential off-target sites—whereas the sgRNA2R did not. All fourteen F1 offspring were also detected for the off-target mutations, five of them carried both the target mutation and off-target mutation, and six of them carried the target mutation but not the off-target mutation; thus, the off-target mutation could be omitted in the offspring.

In this study, we found that the *ATP7B* point mutation or knock-out rabbits exhibited defects and abnormalities in ceruloplasmin, copper in plasma, copper in tissues, morphology of liver tissue and indexes of correlation with liver function. These results suggest that mutation of *ATP7B* is insufficient for copper transport and metabolism *in vivo*, in agreement with findings from previous reports in humans^46,47^.

Ceruloplasmin is mainly synthesized in the liver and functions as a major carrier of copper in the blood. Using ceruloplasmin to identify patients with WD is further complicated by overlap with certain heterozygotes, whereas 20% of heterozygotes have decreased levels of ceruloplasmin. The ELISA results showed that the concentration of ceruloplasmin in KO and point mutant rabbits was lower than that in WT rabbits, in agreement with previous findings in humans^48^. More significantly, we found a similar ratio of copper content in the liver between rabbits and humans at the onset of WD. There was a nine-fold increase in the mutant rabbits and a five-fold increase in humans compared with an approximately 30-fold increase in mice^49^.

These rabbits died of WD in approximately three months. However, in humans, the onset of WD is at 5 to 50 years old. This significant difference may be attributed to two reasons: human WD is derived from different types of mutations and different WD patients exhibit varying levels of copper intake because they live in different regions and lead different lifestyles. All WD rabbits may have died early, before adolescence, because they carried the same mutation, had a similar genetic background, and lived on the same diet and because there is no cure for hepatitis. These features of the WD rabbit make it a potential research model for treatment testing. Therefore, more work should be conducted on this model in the future.

In summary, this is a successful study to build a precision point mutation rabbit disease model using CRISPR/Cas9 technology following gene knockout^17,50^ and gene knock-in^33^ in rabbits. The present study developed a potential rabbit model for WD disease. Further validation of the model will be required to determine whether it is suitable for gene therapy research.

## Electronic supplementary material

Supplementary Information
