# Supplementary material for: Production of Wilson Disease Model Rabbits with Homology-Directed Precision Point Mutations in the ATP7B Gene Using the CRISPR/Cas9 System
Source: Sci Rep. 2018 Jan 22;8:1332. doi: 10.1038/s41598-018-19774-4 (PMC5778067; doi:10.1038/s41598-018-19774-4)
Supplement: Supplementary file 1 — Supplementary Information [file 41598_2018_19774_MOESM1_ESM.pdf]

Title

Production of Wilson Disease Model Rabbits with Homology-Directed Precision Point Mutations in the *ATP7B* Gene Using the CRISPR/Cas9 System

Weihua Jiang[1#], Lili Liu[1#], Qiurong Chang[1], Fengying Xing[1], Zhengwen Ma[1], Zhenfu Fang[1], Jing Zhou[1], Li Fu[1], Huiyang Wang[1], Xingxu Huang[2], Xuejin Chen[1], Yao Li[1]& Shangang Li[1]

1 Department of Laboratory Animal Science, School of Medicine, Shanghai Jiao Tong University, 200025 Shanghai, China.

2 School of Life Science and Technology, Shanghai Tech University, 100 Haike Rd., Pudong New Area, Shanghai, 201210, China.

Supplementary-information

Supplementary TableS1

Supplementary TableS2

Supplementary Fig.S1

Supplementary Fig.S2

Supplementary Fig.S3

Supplementary Fig.S4

Supplementary Fig.S5

Supplementary Fig.S6

Supplementary Fig.S7

Supplementary Fig.S8

Full -length gel of different figures in the article

Supplementary TableS1 PCR primers

Name of Primers	Sequence（5’-3’）
P-sgRNA1F	TTAATACGACTCACTATAGGTTCATTTCCCTGGGGCGGGTTTTAGAGCTAGAAATAGC
P-sgRNA2R	TTAATACGACTCACTATAGGTTCCAGCCACCGCCCCAGTTTTAGAGCTAGAAATAGC
ssODN	TGACATTCTTCGACACGCCGCCCATGCTGTTTGTGTTCATTTCCCTAGGGCTGTGGCTGGAACACGTGGCAAAGGTAACTGCAGCTCTAGGTTAAGAA
T7R1	AAAAGCACCGACTCGGTGCCA
T7 primer	GAAATTAATACGACTCACTATA
P-sgF0F	GTTTTAGAGCTAGAAATAGC
rATP7BJDF	TTGGCAGCCTTTATTCTTTGTTTT
rATP7BJDR	AGACCTGTGGGCTCCGACTTGAA

Supplementary Table S2 Potential off target sequences (POTS) for sgRNA1F and sgRNA2R. The potential off-target sites of the two sgRNAs were predicted using the online-based tool Cas9 (http://cas9.wicp.net)(not access now) and were confirmed by another online-based tool (http://crispr.mit.edu/). The top three POTS were selected for each sgRNA according to the ranking scores. The blue colored nts are the sgRNA sequences, the red nts in square frame are the PAMs, and the red nts with underline is the primers used for PCR and sequence analysis.

sgRNA1F<br><br>potential off<br><br>target #1	Oryctolagus cuniculus chr15 genomic scaffold, OryCun2.0<br>NCBI Reference Sequence: NW_003159296.1 55548211- 55548193,1<br>Not in gene<br>AAAACATTTTGGGCTGACCTGGGGGGGACGGGCGGAAAGGAGAAAGAACTCTCCTGTT<br>TTCTGGTGCTGAACCTTGCGGCTGCAGTGTAAGAGCTGAGCCCCTCCGCTCACTGCGCC<br>AGTAGTTAGAAGAAGCAAACTGAAGACTGCTTCTCCCCGCAGTGAGTGCCGTCTTCGTG<br>CGAGCACCTGGCCTAGCCGCACCTCTCAGAGCCGGTGGCCCAGAACAGCTGTTTGAACG<br>CGCTTGCTCTTTCTTTTCCTTCTAATGAGGAACCTCCTTTCCCTGGGGCGGGGGTGGGAG<br>TGGATCTCTCTATTGACGTCTGCCTCTTTCCTTTCTAAGACAGAAAACCAGTGCAAGGCCA<br>CTATTGGGGCACAGCAGTTTAGGCTACTGCTTGCAACACCTGGATCTCATAGCAAAGCAC<br>TGGTTCGAAGCCTGGCTGCCCTACTTTGGATCCAGCTCCCTGCTAATGCACCTGGGCAGG<br>CAGAGGAAGATGGCGCAAGTCCCTGAGCTCCTATCACCCACATGCGAGACCCAGACAGT<br>GTTCTGGACTCCTGGTTTCACCCTGACCCAGGCCAGGCTGCTGTGGGTCTTTGGGGAGC<br>GAACCAGCAAATGAAGATCTCCCTCTCACTCGCTCTCCCTCTACTCTACCTTTCAAATCAAT<br>CAATCAAATACAAATAAATCTTTAAAAGGGAAA
sgRNA1F<br><br>potential off<br><br>target #2	Oryctolagus cuniculus chr3 genomic scaffold, OryCun2.0<br>GenBank: GL018611.1 25052878- 25052894,1<br>Not in gene<br>CCAATGTCTTTCTTTTTTTTTTTTATTTCATCACCCAAACATGCACGATGCTGCTTTGAGCT<br>GTTGTCTCCTACCTTCCTATGTCTGGAAATTGAATTACTAATCATTTTGTCATTGCTTAGTCA<br>AAATGTCCACATTGCAATGTGTGGTGCGTGTTCTACCCAGAATTTAACTGGACAAGACAA<br>GGAGAGAAGCTGCAGGGTATTTGGTCATGCAAGATAAAAGCCTGTTATTGTCCTAATGGC<br>ATGGAGCAGGCCTTCCTAAGAGGGGGAAAGCAGCTGCCATGGCAACAGGTTCTGTTTTC<br>CCTGGGGCGGGGGTGGGGGTAGGGGGAGCTTGTGTCGCGCTGATAGCCCTGGGGTGT<br>GGAGAGCGTACATCACAAGCCAGCAGTGACAGTACCGTCTGGCGCACTGAGAGCAGTT<br>GCTGCCTCATAGAGTGGAGATTATATATGAGTTGGTGGGTTGGTGCTGGCTTTGTGTCTG<br>CCTGGCACAGAGAGCTGCGGCACCTCAGAACCATAGGTAAAGGGCAATAGATGGGCTCT<br>GGAGCCCTATGTGTCCCTAGCAGAGAAGCAGCCAGCAAGATGGCCCTGTGCCAGCCCCC<br>GAAGAAGCCTGCACCTCTGGCTTGGGGCTCCTCCTAGTCAACCTCAGTGTTCCACTCG

sgRNA1F potential off target #3	Oryctolagus cuniculus chr3 genomic scaffold, OryCun2.0<br>NCBI Reference Sequence:.111401845- 11401831,-1<br>Not in gene<br>GGAACATCCTCTTGACACGCCAGCAAGGAATTCGATCCTAATCTCGGAGCAGTTGAC<br>AGCCCTCTGAATGGAATAAATCTTGGCTTCATGAAATATTCAGCGTCCTCTTTTACATTT<br>TGCTTTGTCCTCCCGTTCCTGATGGTGGAAATGTTATCATAAAGCAACAGTGAGCCTCC<br>GGCTGATGTGGGGAGCAGGAAGACAGTAAAGCCTTTGGCTGGACTTCCCTTGTTGC<br>CAGTCCCCATCAGCTCAACACCCTTGCCCGTCTTTGCTCTTTGTGGGAGAACTGGTCT<br>ACCTGGACCCTGTGGCTTGCGGTGGGCTCAGCCCTGGGGGAAAAACAGACAGGAC<br>AGCAGAGGGTGAGGAGAGAGAAGGTGAGGCTTCTTACTCCACTGATCCGCTGTGCC<br>CACCGCCCCAGGGAGGGCCTGGCAGTGTGTGCATGTCTGTACTCCACTTTCTTCCTA<br>GATGTATCCTCACATACTCTCCTAACCCGTCCCCATACCTCTTCCATGAAGACAAGGCA<br>AAGAGCTCTGCCACCTGTTCAGAGCTGATGCCCTTGAACTTGAGCTCCCCCTTGGTCC<br>TCAGTCATGGCACTGATATGTCCACATGTGTCCAGCTAGGGCCTAGTCAACACTTGATT<br>CGGTTACACATGGAAGTGCTGAGGCCCATCCCAGCGAAGCAGCTGGCCCAAAGTAG<br>CCTTGCCTCGAAGAATCCCGTCCAATACAAAGCATGGGGTCCCGGTCACAAGCAGAG<br>TGCCCATTAATCAGTGCTTGCCGCACCCTCCATAGCCAGCCCCTGCCCAGGCCTGACG<br>ATGGGCTCCT
sgRNA2R potential off target #1	Oryctolagus cuniculus chr4 genomic scaffold, OryCun2.0<br>NCBI Reference Sequence: GL018619.1 33227744-33227764 ,+1<br>In gene zinc finger protein 703-B-like, in Exon.<br>CTGACGAGGAGGAGGGGCAGCCCCCAAGGGCCTGGTGGTGTTTGCAGACCTTGTC<br>CATCACCGGGCCTCAGAGGAAACCTGCACCTTGGCTTTCATCGTGCAGAGGAGAGCT<br>GAGGCTTTGCCAGGCGGCGCTCAGCCCACCGAGTGAAGGAAGGAGAGTCCAGCCG<br>GGCTCTGCCCCTGTTCTACCTGCCCTGTGGCTGTGCAGCGGGGAAAGCAGACACACC<br>AGGTCCGGGAGGCCACGTCTGACCATGAGAAGGAGAGTGCCGGGGTTGGGAGGCA<br>GGTCCCACCCACCGCCCCAGGGAGGACTCTCTTCTCGGTGGCATTTATCTCATGCATC<br>AGCCGCTCCTATTTCTTGCAATAATTCATCCAATATATCTTCCTTAGCAGACTAACAGCT<br>CCACCAGCATCGGGAACCAAGTCTGCCTTCCACCCTGGTGCCTGGCACACAGGAGGT<br>GTTCTGTAAAGTACTGGTTGAAAAATAAATGAATGAATCAGCCTCTTCATGCAAAGAG<br>GAAGATCAGCAAATAGGAGGGTCACGCTTTCCATGGGGGAAGCCCTTGGCAAGCTT<br>CAAAAGAAGTGAGCCCGTTGGTAGAGGCGATCA
sgRNA2R potential off target #2	Oryctolagus cuniculus chr1 genomic scaffold, OryCun2.0<br>NCBI Reference Sequence: NC_013669.1 18776125- 18776106,-1<br>In gene FAM214B, in Exon.<br>TGAGGATGAGGGTCCAGCCCCTCGGAGGCGCCGGGGAACCCTGGGCCACCCTCCT<br>GCTGCCAACAGTTCTGATGCCAAAGCCACACCCTTCTGGAGTCACCTGCTGCCTGGA<br>CCCAAGGAGCCTGTTTTGGTGGTAAGCCAATACCACCGTGAAGTGGATACTTGTGT<br>GGGTGAAGGTGGAGGACTGGGGCGATGCCTCTTGTAAAGCCTCTGATGAGCTCCT<br>GTCCCAAAATACACCTGGCCTCCACCCAGTCTCCTTTCTTAACCTTTCACCTCTATAG<br>GACCCAACAGACTGCAGTCCCATGGGGCGGAGGCTGAAAGGTGCCCGTCGCCTGA<br>AGCTGTAAGTGACCAGCTTCTCCCTCTGTCCTGTTCCTGAGAACGGGGGTCAAAATC<br>TATAGTCCAGGATAGGGGACCCCAGAGCAGGAGCCCTAGATAATGGCAAACAATG<br>GAACCCGAGGACATGGGCAGGAATGCCGGAATATCTCAAGCTATGCATCCCTTCCT<br>CCACCAGGAGCTCCCTTCGAAGCCTCCGGAAGAGCCCAGGCCTGCTGAGCCCCCCC<br>AGTGCCTCCCCTGTTCCTACCCCTGCTGTCAGCCGTACCCTGCTGGGCAACTTTGAG<br>GTA

sgRNA2R potential off target #3	Oryctolagus cuniculus chrUn0937 genomic scaffold, OryCun2.0<br>NCBI Reference Sequence: NC_013690.1 34368- 34349,-1<br>Not in gene<br>AGGTCGCCGCGGAACCACGTCCTCCCGTCACCAAAAGAGCCAAGGCAGCCACCCGC<br>TCGCGTCCCGAAGGCACGAGCCTGAGTACGCAGGCGCGCTGGCTGCTGCCGAGGC<br>CGCTTCCGGGTCACGTGGCCCAACTTCCGGTTCGCACCACTCCTCTCGGTGACGACG<br>CGGCCAATCGGTTTCGTCGGACTACGCGGTCGCCTGCGTCGCCTCCGGGTGCCGTT<br>GGCTGCCGTTGGGCCGCTTCCCGCGCGTCGTCCAGCCCGCCTCTCCGCTAGCCGCA<br>GCCCCAGCTCCTCCCTCGTCCGGCCCCTGGGGCGGAGTCTGTTGGCTCGCCCTCCCC<br>TCGCCGCCTAAGCCTGCTTTCCGACAACTGGGTCCCCTCCCCACAGCAACACGGCGT<br>CTCCTACTCGCCGGCGGGGACAGGGGGTTTCCCCCTCAGCCGCCGCCGCTGCCGGC<br>CGAGCCCAGCCGCACCCGCGGCCCGCGGCTGCCGTAAGTCCCGAGGGCGAGTGGG<br>GACGCTGGGGATGGGGATGGGGAGGGGGAGGGATGGCTGGGCGCGCCCCCCGC<br>CGCCCCGCGCGCGCTCCGACGACCCCACCCCGAGCCCGCCGGCCCGCCACCTGGGC<br>GCCGCGC

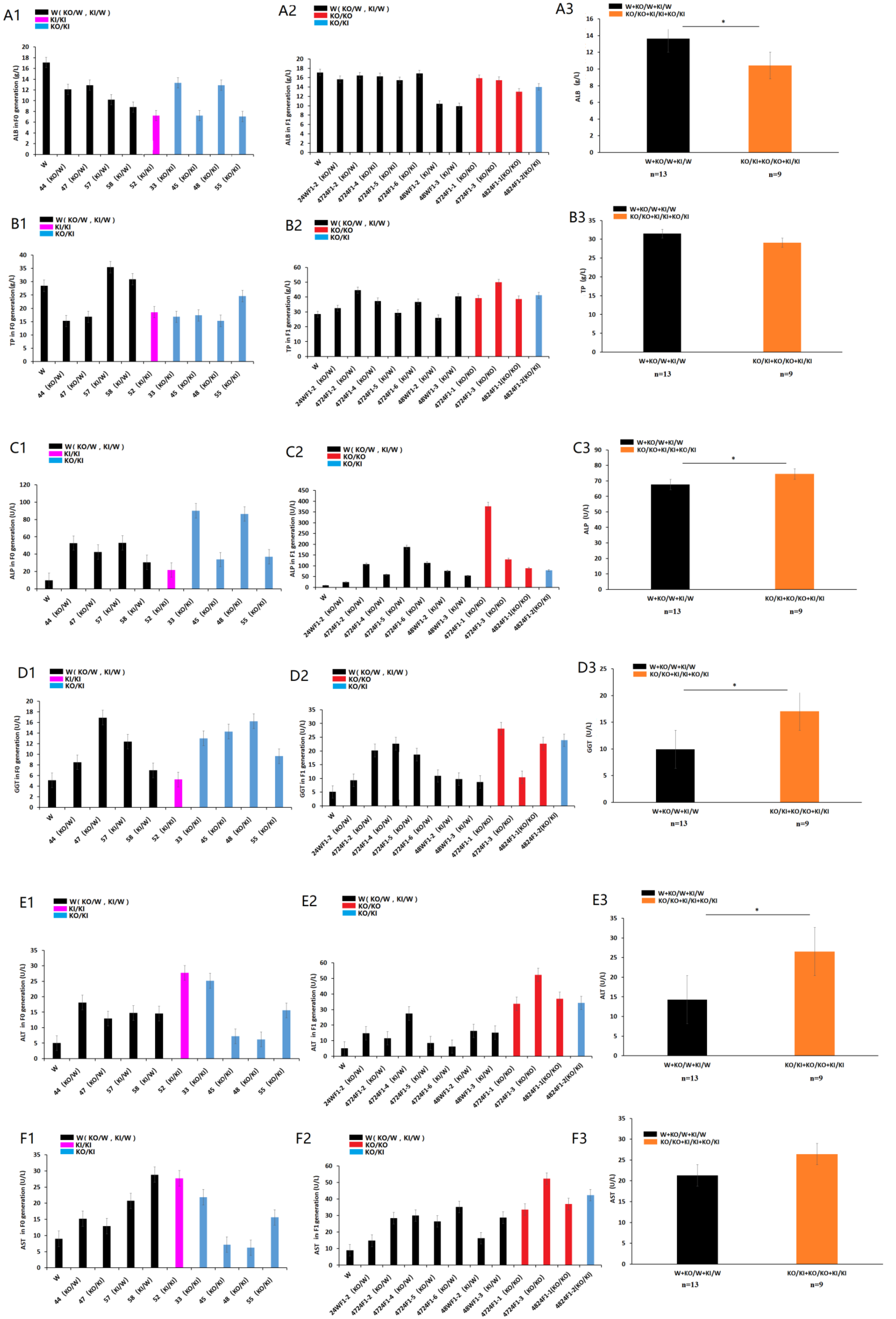

A1
W(KO/W , KI/W)
KI/KI
KO/KI
ALB in F0 generation (g/L)
W
44 (KO/W)
47 (KO/W)
57 (KI/W)
58 (KI/W)
52 (KI/KI)
33 (KO/KI)
45 (KO/KI)
48 (KO/KI)
55 (KO/KI)
A2
W(KO/W , KI/W)
KO/KO
KO/KI
ALB in F1 generation (g/L)
W
24WF1-2 (KO/W)
4724F1-2 (KO/W)
4724F1-4 (KO/KI)
4724F1-5 (KO/KI)
4724F1-6 (KO/KI)
48WF1-2 (KI/W)
48WF1-3 (KI/W)
4724F1-1 (KO/KO)
4724F1-3 (KO/KO)
4824F1-1(KO/KO)
4824F1-2(KO/KI)
A3
W+KO/W+KI/W
KO/KO+KI/KI+KO/KI
ALB (g/L)
W+KO/W+KI/W
n=13
KO/KI+KO/KI+KI/KI
n=9
B1
TP in F0 generation(g/L)
B2
TP in F1 generation (g/L)
B3
TP (g/L)
KO/KI+KO/KO+KI/KI
C1
ALP in F0 generation (U/L)
C2
ALP in F1 generation (U/L)
C3
ALP (U/L)
D1
GGT in F0 generation (U/L)
D2
GGT in F1 generation (U/L)
D3
GGT (U/L)
E1
ALT in F0 generation (U/L)
E2
ALT in F1 generation (U/L)
E3
ALT (U/L)
F1
AST in F0 generation (U/L)
F2
AST in F1 generation (U/L)
F3
AST (U/L)

Fig. S1 Levels of liver function indicators in the *ATP7B* mutated rabbits

(A1) The plasma ALB level in the *ATP7B* mutated rabbits of F0, (A2) The plasma ALB level in the *ATP7B* mutated rabbits of F1, (A3) Differences of plasma ALB level between the homozygous and WT (*P<0.05) in all F0 and F1 rabbits. (B1) The plasma TP level in the *ATP7B* mutated rabbits of F0. (B2) The plasma TP level in the *ATP7B* mutated rabbits of F1. (B3) Differences of plasma TP level between the the homozygous and WT (P>0.05) in all F0 and F1 rabbits . (C1) The plasma ALP level in the *ATP7B* mutated rabbits of F0, (C2) The plasma ALP level in the *ATP7B* mutated rabbits of F1, (C3) Differences of plasma ALP level between the homozygous and WT is significant (*P<0.05) in all F0 and F1 rabbits. (D1) The plasma GGT level in the *ATP7B* mutated rabbits of F0, (D2) The plasma GGT level in the *ATP7B* mutated rabbits of F1, (D3) Differences of plasma GGT level between the homozygous and WT individuals is significant (*P<0.05) in all F0 and F1 rabbits. (E1) The plasma ALT level in the *ATP7B* mutated rabbits of F0, (E2) The plasma ALT level in the *ATP7B* mutated rabbits of F1, (E3) Differences of plasma ALT level between the homozygous and wild-type individuals is significant (*P<0.05) in all F0 and F1 rabbits . (F1) The plasma AST level in the *ATP7B* mutated rabbits of F0, (F2) The plasma AST level in the *ATP7B* mutated rabbits of F1, (F3) Differences of plasma AST level between the homozygous and WT individuals is significant (P>0.05) in all F0 and F1 rabbits. Homozygous includes double KO,double KI and KI/KO, WT in cludes wild type and W/KO: W/KI.

Species | Site | Amino acid sequence | Identities 730/1453(50%) | Positives 852/1453(58%) | Gaps 393/1453(27%) | Site

```
Rat    2     RKVTAK--ASRKI----SKATR-W-GSMKSA---DNVGYGG--DSTCSSST--TTGVVSI  46
             R++TA+  ASRKI    S  TR W  +MK +   DNVGY G  D    SS   T+ V  +
Human  6     RQITAREGASRKILSKLSLPTRAWEPAMKKSFAFDNVGYEGGLDGLGPSSQVATSTVRIL  65

Rat    47    GMTCHSCVKSI-DRISS-KGIVSIKVS---GSATVKYVSVNICI--------DMGASAA-  92
             GMTC SCVKSI DRIS+ KGI+S+KVS   GSATVKYV   +C+        DMG  A+
Human  66    GMTCQSCVKSIEDRISNLKGIISMKVSLEQGSATVKYVPSVVCLQQVCHQIGDMGFEASI  125

Rat    93    --GKAASW-SRS---SAAVVK-RV-GMTC-SCVSSI-GKIRK--GVVRVKVS---SNAVI  137
               GKAASW SRS     AVVK RV GMTC SCVSSI GK+RK  GVVRVKVS     AVI
Human  126   AEGKAASWPSRSLPAQEAVVKLRVEGMTCQSCVSSIEGKVRKLQGVVRVKVSLSNQEAVI  185

Rat    138   TY--YI-----DRDHICDMG--AAIKNRTAR----GIDINK--STNKRAAVINSNHTGHN  182
             TY  Y+      RDH+ DMG  AAIK++ A      IDI +  STN +  + ++N   +N
Human  186   TYQPYLIQPEDLRDHVNDMGFEAAIKSKVAPLSLGPIDIERLQSTNPKRPLSSANQNFNN  245

Rat    183   HAT-------------RIDGMHCKSCV-----NIGNIGGVN---IHVSNKTAV--YDSSC  219
               T             RIDGMHCKSCV     NIG + GV    + + NKTA   YD SC
Human  246   SETLGHQGSHVVTLQLRIDGMHCKSCVLNIEENIGQLLGVQSIQVSLENKTAQVKYDPSC  305

Rat    220   ITTAIA-----------GYKVS--DGKSGS-----------------SSVSGSSRGCRTA  249
              T+ +A            +KVS  DG  GS                 + V G+   C T
Human  306   -TSPVALQRAIEALPPGNFKVSLPDGAEGSGTDHRSSSSHSPGSPPRNQVQGT---CSTT  361

Rat    250   -VTITGIRDSSV------MDMSMKGVIDIS----AGTGAVY--DSVVSSD--RTAV-DMG  293
              + I G+  +S       M   ++GV  IS     GT  V    SV+S +  R A+ DMG
Human  362   LIAIAGMTCASCVHSIEGMISQLEGVQQISVSLAEGTATVLYNPSVISPEELRAAIEDMG  421

Rat    294   VSV----NNITTNRV---SSGNSVAVGDSGSVNMASDTRGTHGG----------YSDSSG  336
                     + +TN +   S+GNS+     G+     +    H G           + S
Human  422   FEASVVSESCSTNPLGNHSAGNSMVQTTDGTPTSVQEV-APHTGRLPANHAPDILAKSPQ  480

Rat    337   GT----ASKCV--IKGMTCASCVSNIR---SRHAGIS---VVAMSGKA-VKYD--VISRI  381
              T      KC   IKGMTCASCVSNI     + AG+    V  M+GKA +KYD  VI  +
Human  481   STRAVAPQKCFLQIKGMTCASCVSNIERNLQKEAGVLSVLVALMAGKAEIKYDPEVIQPL  540

Rat    382   AI--------DGAAIMDNTVSGD----IIITGMTCASCVHNISK--TRTNGITYASVA-A  426
              I          AA+M++    D    + ITGMTCASCVHNI    TRTNGITYASVA A
Human  541   EIAQFIQDLGFEAAVMEDYAGSDGNIELTITGMTCASCVHNIESKLTRTNGITYASVALA  600

Rat    427   TSKAHVK---DIIG-RDIIKVI--IG-HAS-AHRN-NAHH-DHKTI--KWKKSCSVGIVM  474
             TSKA VK   +IIG RDIIK+I  IG HAS A RN NAHH DHK    +WKKS    +V
Human  601   TSKALVKFDPEIIGPRDIIKIIEEIGFHASLAQRNPNAHHLDHKMEIKQWKKSFLCSLVF  660

Rat    475   G------MIYMI----SSKHTMV-DHNIIGSVNII-------CT----VGGWYYV--AYK  510
             G      MIYM+        +MV DHNII  ++I+       CT    +GGWY+   AYK
Human  661   GIPVMALMIYMLIPSNEPHQSMVLDHNIIPGLSILNLIFFILCTFVQLLGGWYFYVQAYK  720

Rat    511   S-RHKSANMDVIV--ATTIAYAYSVIVVAIAKAK-------SVTDT------MVIAGRW-  553
             S RH+SANMDV++  AT+IAY YS++++ +A A+       +  DT       +  GRW
Human  721   SLRHRSANMDVLIVLATSIAYVYSLVILVVAVAEKAERSPVTFFDTPPMLFVFIALGRWL  780

Rat    554   -HVAKSKTSAA-----KMSAT-ATVVT--GDNI---------RVMVRGDIIKVVGG---K  592
              H+AKSKTS A      + AT ATVVT   DN+          ++ RGDI+KVV G
Human  781   EHLAKSKTSEALAKLMSLQATEATVVTLGEDNLIIREEQVPMELVQRGDIVKVVPGGKFP  840

Rat    593   VDGKV--GNTMADS--ITG-AM-VTKK-GSIVIAGSINAHGSV-IKATHVGNDTT--AIV  642
             VDGKV  GNTMAD   ITG AM VTKK GS VIAGSINAHGSV IKATHVGNDTT   IV
Human  841   VDGKVLEGNTMADESLITGEAMPVTKKPGSTVIAGSINAHGSVLIKATHVGNDTTLAQIV  900

Rat    643   KV----AMSKA----IADR-SGYV---IIIISTT--VVWIIIGV--DGIVKY----SSKH  682
             K+     MSKA    +ADR SGY    III+ST   VVWI+IG    G+V+      +KH
Human  901   KLVEEAQMSKAPIQQLADRFSGYFVPFIIIMSTLTLVVWIVIGFIDFGVVQRYFPNPNKH  960

Rat    683   IST--VIIR---ATSITV-CIAC-CS---GATTAVMVGTGVAA-NGV-IKGGK---MAHK  727
             IS   VIIR    TSITV CIAC CS      TAVMVGTGVAA NG+ IKGGK   MAHK
Human  961   ISQTEVIIRFAFQTSITVLCIACPCSLGLATPTAVMVGTGVAAQNGILIKGGKPLEMAHK  1020

Rat    728   IKTVM-DKTGTITHGV-RVMRV----DVATS--RKV-AVVGTA--ASSH--GVAVTKYCK  774
             IKTVM DKTGTITHGV RVMRV    DVAT   RKV AVVGTA  +S H  GVAVTKYCK
Human  1021  IKTVMFDKTGTITHGVPRVMRVLLLGDVATLPLRKVLAVVGTAEASSEHPLGVAVTKYCK  1080

Rat    775   G-----TTGYSTD---AVGCGISCKVSNVS--IAHR------GTAHIGVGN--------I  810
                   T GY TD     GCGI CKVSNV   +AH         +H+            +
Human  1081  EELGTETLGYCTDFQAVPGCGIGCKVSNVEGILAHSERPLSAPASHLNEAGSLPAEKDAV  1140

Rat    811   GGTGTSVIGNR-WMRRNG-TISSDISDAMTDH-MKG-TAI-VAIDGV-CGMIAIADAVK-  863
               T + +IGNR W+RRNG TISSD+SDAMTDH MKG TAI VAIDGV CGMIAIADAVK
Human  1141  PQTFSVLIGNREWLRRNGLTISSDVSDAMTDHEMKGQTAILVAIDGVLCGMIAIADAVKQ  1200

Rat    864   --AAAIYT-KSMGVDV-AITGDNRKTARAIAT-VGINKV----AVSHKVAKV----NKGK  910
               A A++T +SMGVDV  ITGDNRKTARAIAT VGINKV      SHKVAKV    NKGK
Human  1201  EAALAVHTLQSMGVDVVLITGDNRKTARAIATQVGINKVFAEVLPSHKVAKVQELQNKGK  1260

Rat    911   KVAMVGDGVNDSAA---ADVGIAIGTGTDVAI-AADVV-IRND--DVVASIH-SKRTVRR  962
             KVAMVGDGVNDS A   AD+G+AIGTGTDVAI AADVV IRND  DVVASIH SKRTVRR
Human  1261  KVAMVGDGVNDSPALAQADMGVAIGTGTDVAIEAADVVLIRNDLLDVVASIHLSKRTVRR  1320

Rat    963   IRVNV---AIYNMVGI-IAAGVM--IGIV---WMG----SAAMAASSVSVVSSKCYRK--  1007
             IR+N+    IYN+VGI IAAGV   IGIV   WMG    +A+  +  +S +  KCY+K
Human  1321  IRINLVLALIYNLVGIPIAAGVFMPIGIVLQPWMGSAAMAASSVSVVLSSLQLKCYKKPD  1380

Rat    1008  -DRYA--AHGRMKS---ASVSVHVGMDDRRRDS-RAT-WD----VSYVSVSSSTSDR-SR  1054
              +RY   AHG MK    + VSVH+GMDDR RDS RAT WD    VS VS+SS TSD+ SR
Human  1381  LERYEAQAHGHMKPLTASQVSVHIGMDDRWRDSPRATPWDQVSYVSQVSLSSLTSDKPSR  1440

Rat    1055  HGGMA-DGGDKWS  1066
             H   A D GDKWS
Human  1441  HSAAADDDGDKWS  1453
```

Fig. S2 Alignment of amino acid sequences encoding human and rat ATP7B protein

```
                                       Identities       Positives       Gaps
Species Site   Amino acid sequence     722/1447(50%)    840/1447(58%)   389/1447(26%)   Site

Mouse  12    RVTAK--ASRKI-------SKAGRWSMKSA---DNVGYGGD----STSSSAATDVVNI-G  54
             ++TA+  ASRKI       ++A   +MK +   DNVGY G       SS  AT  V I G
Human  7     QITAREGASRKILSKLSLPTRAWEPAMKKSFAFDNVGYEGGLDGLGPSSQVATSTVRILG  66

Mouse  55    MTCHSCVKSI-DRISS-KGIVNIKVS---GSATVRYVS----VMNICI---DMGASAA--  100
             MTC SCVKSI DRIS+ KGI+++KVS   GSATV+YV     +  +C    DMG  A+
Human  67    MTCQSCVKSIEDRISNLKGIISMKVSLEQGSATVKYVPSVVCLQQVCHQIGDMGFEASIA  126

Mouse  101   -GKAASW-SRS---SAAVVK-RV-GMTC-SCVSSI-GKIRK--GVVRIKVS---SNAVIT  146
              GKAASW SRS     AVVK RV GMTC SCVSSI GK+RK  GVVR+KVS     AVIT
Human  127   EGKAASWPSRSLPAQEAVVKLRVEGMTCQSCVSSIEGKVRKLQGVVRVKVSLSNQEAVIT  186

Mouse  147   Y--YI-----DRDHICDMG--AAIKNRTAR----GIDVNK--STNKKTVSVISNHT----  187
             Y  Y+      RDH+ DMG  AAIK++ A      ID+ +  STN K     +N
Human  187   YQPYLIQPEDLRDHVNDMGFEAAIKSKVAPLSLGPIDIERLQSTNPKRPLSSANQNFNNS  246

Mouse  188   ---GH-GSY----ATRIDGMHCKSCV-----NIGNIGGVN---IHVSNKTAI--YD-SCV  228
                GH GS+      RIDGMHCKSCV     NIG + GV    + + NKTA   YD SC
Human  247   ETLGHQGSHVVTLQLRIDGMHCKSCVLNIEENIGQLLGVQSIQVSLENKTAQVKYDPSCT  306

Mouse  229   TMTAIA---------GHKVS--DGVNSGSSRHG-------------------GRTAVTIS  258
             +  A+          KVS  DG     + H                      T + I+
Human  307   SPVALQRAIEALPPGNFKVSLPDGAEGSGTDHRSSSSHSPGSPPRNQVQGTCSTTLIAIA  366

Mouse  259   GITCASSVIDMS-----RKGVTSIS----AGTGAVY--DSIVSD---RTAV-DMG-----  298
             G+TCAS V  +       +GV  IS     GT  V    S++S    R A+ DMG
Human  367   GMTCASCVHSIEGMISQLEGVQQISVSLAEGTATVLYNPSVISPEELRAAIEDMGFEASV  426

Mouse  299   VSVNSTTINVRNKSGNSVTMGDIAGSVKMADTRGTHGGH------------SSTSSGATA  346
             VS + +T  + N S  +  +    G+         H G             S  S+ A A
Human  427   VSESCSTNPLGNHSAGNSMVQTTDGTPTSVQEVAPHTGRLPANHAPDILAKSPQSTRAVA  486

Mouse  347   S-KCV--IKGMTCASCVSNIR---SRHAGIS---VVAMSGKA-VKYD--IISRIAI----  390
               KC   IKGMTCASCVSNI     + AG+    V  M+GKA +KYD  +I  + I
Human  487   PQKCFLQIKGMTCASCVSNIERNLQKEAGVLSVLVALMAGKAEIKYDPEVIQPLEIAQFI  546

Mouse  391   ----DGASVMDNTVSGD----IIITGMTCASCVHNISK--TRTNGITYASVA-ATSKAHV  439
                   A+VM++    D    + ITGMTCASCVHNI    TRTNGITYASVA ATSKA V
Human  547   QDLGFEAAVMEDYAGSDGNIELTITGMTCASCVHNIESKLTRTNGITYASVALATSKALV  606

Mouse  440   K---DIVG-RDIIKII--IG-HAS--ARN-NAHH-DHKTI--KWKKSCSVGIVMG-----  481
             K   +I+G RDIIKII  IG HAS   RN NAHH DHK    +WKKS    +V G
Human  607   KFDPEIIGPRDIIKIIEEIGFHASLAQRNPNAHHLDHKMEIKQWKKSFLCSLVFGIPVMA  666

Mouse  482   -MVYMI------SSTTMVDHNIIGSVN-------IICT----VGGWYYV--AYKS-RHRS  520
              M+YM+        + ++DHNII  ++       I+CT    +GGWY+   AYKS RHRS
Human  667   LMIYMLIPSNEPHQSMVLDHNIIPGLSILNLIFFILCTFVQLLGGWYFYVQAYKSLRHRS  726

Mouse  521   ANMDVIV--ATTIAYAYSVIVVAVAKAK-------SVTDT------MVIAGRW--HVAKS  563
             ANMDV++  AT+IAY YS++++ VA A+       +  DT       +  GRW  H+AKS
Human  727   ANMDVLIVLATSIAYVYSLVILVVAVAEKAERSPVTFFDTPPMLFVFIALGRWLEHLAKS  786

Mouse  564   KTSAA-----KMSAT-ATVVT--GDNI---------RVMVRGDVIKVVGG---KVDGKV-  602
             KTS A      + AT ATVVT   DN+          ++ RGD++KVV G    VDGKV
Human  787   KTSEALAKLMSLQATEATVVTLGEDNLIIREEQVPMELVQRGDIVKVVPGGKFPVDGKVL  846

Mouse  603   -GNTMADS--ITG-AM-VTKK-GSIVIAGSINAHGSV--KATHVGNDTT--AIVKV----  648
              GNTMAD   ITG AM VTKK GS VIAGSINAHGSV  KATHVGNDTT   IVK+
Human  847   EGNTMADESLITGEAMPVTKKPGSTVIAGSINAHGSVLIKATHVGNDTTLAQIVKLVEEA  906

Mouse  649   AMSKA----IADR-SGY---VIIIIS--TTVVWIVIGV--DGVVKY----SSKHIST--V  690
              MSKA    +ADR SGY    III+S  T VVWIVIG    GVV+      +KHIS   V
Human  907   QMSKAPIQQLADRFSGYFVPFIIIMSTLTLVVWIVIGFIDFGVVQRYFPNPNKHISQTEV  966

Mouse  691   IIR---ATSITV-CIAC-CS---GATTAVMVGTGVAA-NGV-IKGGK---MAHKIKTVM-  736
             IIR    TSITV CIAC CS      TAVMVGTGVAA NG+ IKGGK   MAHKIKTVM
Human  967   IIRFAFQTSITVLCIACPCSLGLATPTAVMVGTGVAAQNGILIKGGKPLEMAHKIKTVMF  1026

Mouse  737   DKTGTITHGV-RVMRA----DVAT---RKV-AVVGT--AASSH--GVAVTKYCK-----G  778
             DKTGTITHGV RVMR     DVAT   RKV AVVGT  A+S H  GVAVTKYCK
Human  1027  DKTGTITHGVPRVMRVLLLGDVATLPLRKVLAVVGTAEASSEHPLGVAVTKYCKEELGTE  1086

Mouse  779   TTGYSTD---AVGCGISCKVSNV-GI----ARSDTAHVGVGNTGGAGTS-----------  819
             T GY TD     GCGI CKVSNV GI     R  +A     N  G+  +
Human  1087  TLGYCTDFQAVPGCGIGCKVSNVEGILAHSERPLSAPASHLNEAGSLPAEKDAVPQTFSV  1146

Mouse  820   VIGNR-WMRRNG-TISSDISDAMTDH-MKG-TAI-VAIDGV-CGMIAIADAVK---AAAI  870
             +IGNR W+RRNG TISSD+SDAMTDH MKG TAI VAIDGV CGMIAIADAVK   A A+
Human  1147  LIGNREWLRRNGLTISSDVSDAMTDHEMKGQTAILVAIDGVLCGMIAIADAVKQEAALAV  1206

Mouse  871   YT-KSMGVD-VAITGDNRKTARAIAT-VGINK----VAVSHKVAKVN-----GKKVAMVG  918
             +T +SMGVD V ITGDNRKTARAIAT VGINK    V  SHKVAKV      GKKVAMVG
Human  1207  HTLQSMGVDVVLITGDNRKTARAIATQVGINKVFAEVLPSHKVAKVQELQNKGKKVAMVG  1266

Mouse  919   DGVNDSAA---ADVGIAIGTGTDVAI-AADVV-IRND--DVVASIH-SKRTVRRIRVNV-  969
             DGVNDS A   AD+G+AIGTGTDVAI AADVV IRND  DVVASIH SKRTVRRIR+N+
Human  1267  DGVNDSPALAQADMGVAIGTGTDVAIEAADVVLIRNDLLDVVASIHLSKRTVRRIRINLV  1326

Mouse  970   --AIYNMVGI-IAAGVM--IGIV---WMG----SAAMAASSVSVVSSKCYRK---DRYA-  1013
                IYN+VGI IAAGV   IGIV   WMG    +A+  +  +S +  KCY+K   +RY
Human  1327  LALIYNLVGIPIAAGVFMPIGIVLQPWMGSAAMAASSVSVVLSSLQLKCYKKPDLERYEA  1386

Mouse  1014  -AHGRMKS---ASVSVHIGMDDRRRDS-RATAWD----VSYVSVSSSTSDR-SRHGGAA-  1062
              AHG MK    + VSVHIGMDDR RDS RAT WD    VS VS+SS TSD+ SRH  AA
Human  1387  QAHGHMKPLTASQVSVHIGMDDRWRDSPRATPWDQVSYVSQVSLSSLTSDKPSRHSAAAD  1446

Mouse  1063  DGGDKWS  1069
             D GDKWS
Human  1447  DDGDKWS  1453
```

Fig. S3 Alignment of amino acid sequences encoding human and mouse ATP7B protein

Identities 1278/1466(87%) | Positives 1358/1466(92%) | Gaps 2/1466(0%)

```
Species  Site   Amino acid sequence                                            Site

Human    18     ILSKLSLPTRAWEPAMKKSFAFDNVGYEGGLDGLGPSSQVATSTVRILGMTCQSCVKSIE   77
                ILSKLSLP RAWE AMK+SFAFDNVGYEGGLD + PS    T  V ILGMTCQSCVKSIE
Rabbit   21     ILSKLSLPARAWEAAMKQSFAFDNVGYEGGLDSMCPSP-TTTGIVSILGMTCQSCVKSIE   79

Human    78     DRISNLKGIISMKVSLEQGSATVKYVPSVVCLQQVCHQIGDMGFEASIAEGKAASWPSRS   137
                DRIS+LKGI+S+K+SLEQ SATVKYVPSV+ LQQVCH IGDMG+EAS+ EGKAASWPSRS
Rabbit   80     DRISSLKGIVSIKISLEQASATVKYVPSVMSLQQVCHHIGDMGYEASVTEGKAASWPSRS   139

Human    138    LPAQEAVVKLRVEGMTCQSCVSSIEGKVRKLQGVVRVKVSLSNQEAVITYQPYLIQPEDL   197
                LPAQEAV+KLRVEGMTCQSCVSSIEGK+ KLQGVVRV+VSL NQEAVITYQPYLIQPEDL
Rabbit   140    LPAQEAVIKLRVEGMTCQSCVSSIEGKIGKLQGVVRVRVSLGNQEAVITYQPYLIQPEDL   199

Human    198    RDHVNDMGFEAAIKSKVAPLSLGPIDIERLQSTNPKRPLSSANQNFNNSETLGHQGSHVV   257
                RDHVNDMGFEA IK+K+APLSLGPIDIERLQ+TN KRP  S NQN NNSET  H GS V
Rabbit   200    RDHVNDMGFEATIKNKMAPLSLGPIDIERLQNTNLKRPSVSTNQNLNNSETSAHPGSQVA   259

Human    258    TLQLRIDGMHCKSCVLNIEENIGQLLGVQSIQVSLENKTAQVKYDPSCTSPVALQRAIEA   317
                TLQLR+DGMHCKSCVLNIE NIGQL GVQ+IQV LEN+TAQV+YDPS  +P +LQ+AIEA
Rabbit   260    TLQLRVDGMHCKSCVLNIEGNIGQLPGVQNIQVFLENRTAQVQYDPSHVTPESLQKAIEA   319

Human    318    LPPGNFKVSLPDGAEGSGTDHRSSSSHSPGSPPRNQVQGTCSTTLIAIAGMTCASCVHSI   377
                LPPGNFKVSLPDGAE  GT++RSS+ HSP SP R QVQ TCST ++ I GMTCASCV SI
Rabbit   320    LPPGNFKVSLPDGAEERGTENRSSNGHSPVSPQRGQVQSTCSTLVLTIVGMTCASCVQSI   379

Human    378    EGMISQLEGVQQISVSLAEGTATVLYNPAVISPEELRAAIEDMGFEASVVSESCSTNPLG   437
                EG+ISQ EGVQ+ISVSLAEGT TVLY+P+VISPEELRAA+EDMGFEASV+ E+ STN  G
Rabbit   380    EGVISQREGVQRISVSLAEGTGTVLYDPSVISPEELRAAVEDMGFEASVIPENGSTNHTG   439

Human    438    NHSAGNSMVQTTDGTPTSLQEVAPHTGRLPANHAPDILAKSPQSTRAVAPQKCFLQIKGM   497
                NH A NSM     G P S+ ++ PHTG LP NH P   +KSPQST  V PQKCFLQI+GM
Rabbit   440    NHGAENSMAWIKSGAPVSVPDMVPHTGELPKNHHPGRSSKSPQSTGMVVPQKCFLQIRGM   499

Human    498    TCASCVSNIERNLQKEAGVLSVLVALMAGKAEIKYDPEVIQPLEIAQFIQDLGFEAAVME   557
                TCASCVSNIERNLQKE G+LSVLVALMAGKAEIKY+PEVIQP EIAQ IQDLGFEA VME
Rabbit   500    TCASCVSNIERNLQKEDGILSVLVALMAGKAEIKYNPEVIQPPEIAQLIQDLGFEATVME   559

Human    558    DYAGSDGNIELTITGMTCASCVHNIESKLTRTNGITYASVALATSKALVKFDPEIIGPRD   617
                D  GSDG+IEL ITGMTCASCVHNIES LTRTNGITYASVALATSKA VKFDPEIIGPRD
Rabbit   560    DATGSDGDIELIITGMTCASCVHNIESNLTRTNGITYASVALATSKAHVKFDPEIIGPRD   619

Human    618    IIKIIEEIGFHASLAQRNPNAHHLDHKMEIKQWKKSFLCSLVFGIPVMALMIYMLIPSNE   677
                I+KIIEEIGFHASLAQRNPNAHHLDHK+EIKQWKKSFLCSLVFGIPVM LMIYMLIPSN+
Rabbit   620    IVKIIEEIGFHASLAQRNPNAHHLDHKVEIKQWKKSFLCSLVFGIPVMGLMIYMLIPSNQ   679

Human    678    PHQSMVLDHNIIPGLSILNLIFFILCTFVQLLGGWYFYVQAYKSLRHRSANMDVLIVLAT   737
                PH+SM+LDHNIIPGLSILNLIFFILCTFVQ LGGWYFYVQAYKSLRHRSANMDVLIVLAT
Rabbit   680    PHESMILDHNIIPGLSILNLIFFILCTFVQFLGGWYFYVQAYKSLRHRSANMDVLIVLAT   739

Human    738    SIAYVYSLVILVVAVAEKAERSPVTFFDTPPMLFVFIALGRWLEHLAKSKTSEALAKLMS   797
                SIAYVYSL+ILVVAVAE+AERSPVTFFDTPPMLFVFI+LGRWLEH+AKSKTSEALAKLMS
Rabbit   740    SIAYVYSLIILVVAVAEQAERSPVTFFDTPPMLFVFISLGRWLEHVAKSKTSEALAKLMS   799

Human    798    LQATEATVVTLGEDNLIIREEQVPMELVQRGDIVRVVPGGKFPVDGKVLEGNTMADESLI   857
                LQATEATVVTLGEDNLIIREEQVPMELVQRGDI++VVPGGKFPVDGKVLEGNTMADESLI
Rabbit   800    LQATEATVVTLGEDNLIIREEQVPMELVQRGDIIKVVPGGKFPVDGKVLEGNTMADESLI   859

Human    858    TGEAMPVTKKPGSTVIAGSINAHGSVLIKATHVGNDTTLAQIVKLVEEAQMSKAPIQQLA   917
                TGEAMPVTKKPGS VIAGSINAHGSVLI ATHVGNDTTLAQIV+LVEEAQMSKAPIQQLA
Rabbit   860    TGEAMPVTKKPGSIVIAGSINAHGSVLITATHVGNDTTLAQIVRLVEEAQMSKAPIQQLA   919

Human    918    DRFSGYFVPFIIIMSTLTLVVWIVIGFIDFGVVQKYFPNPNKHISQTEVIIRFAFQTSIT   977
                DRFSGYFVPFIII+STLTLVVWI+IGFIDFGVVQKYFPNPNKHISQTE+I+RFAFQTSIT
Rabbit   920    DRFSGYFVPFIIIISTLTLVVWIIIGFIDFGVVQKYFPNPNKHISQTEIILRFAFQTSIT   979

Human    978    VLCIACPCSLGLATPTAVMVGTGVAAQNGILIKGGKPLEMAHKIKTVMFDKTGTITHGVP   1037
                VLCIACPCSLGLATPTAVMVGTGVAAQ+G+LIKGGKPLEMAHKIKTVMFDKTGTITHGVP
Rabbit   980    VLCIACPCSLGLATPTAVMVGTGVAAQHGVLIKGGKPLEMAHKIKTVMFDKTGTITHGVP   1039

Human    1038   RVMRVLLLGDVATLPLRKVLAVVGTAEASSEHPLGVAVTKYCKEELGTETLGYCTDFQAV   1097
                +V+RVLLL D+ATLPLRKVLA+VGTAEASSEHPLG+AVTKYCKEELGTETLGYCTDFQAV
Rabbit   1040   KVLRVLLLVDMATLPLRKVLAMVGTAEASSEHPLGIAVTKYCKEELGTETLGYCTDFQAV   1099

Human    1098   PGCGIGCKVSNVEGILAHSERPLSAPASHLNEAGSLPAEKDAAPQTFSVLIGNREWLRRN   1157
                PGCGIGCKVS+VEG+LAHSER LS  + H+N  GSLPAEKDAA QTF VLIGNREW+RRN
Rabbit   1100   PGCGIGCKVSSVEGLLAHSERLLSEQSGHVNRLGSLPAEKDAASQTFCVLIGNREWMRRN   1159

Human    1158   GLTISSDVSDAMTDHEMKGQTAILVAIDGVLCGMIAIADAVKQEAALAVHTLQSMGVDVV   1217
                GLTISSD++DAMTDHEMKGQTAILVAIDGVLCGMIAIAD+VK EAALAVHTL+SMGVDVV
Rabbit   1160   GLTISSDINDAMTDHEMKGQTAILVAIDGVLCGMIAIADSVKPEAALAVHTLKSMGVDVV   1219

Human    1218   LITGDNRKTARAIATQVGINKVFAEVLPSHKVAKVQELQNKGKKVAMVGDGVNDSPALAQ   1277
                LITGDNRKTARAIATQVGINKVFAEVLPSHKVAKVQELQN+GKKVAMVGDGVNDSPALAQ
Rabbit   1220   LITGDNRKTARAIATQVGINKVFAEVLPSHKVAKVQELQNEGKKVAMVGDGVNDSPALAQ   1279

Human    1278   ADMGVAIGTGTDVAIEAADVVLIRNDLLDVVASIHLSKRTVRRIRINLVLALIYNLVGIP   1337
                AD+G+AIGTGTDVAIEAADVVLIRNDLLDVVASIHLSKRTVRRIR+NLVLALIYNLVGIP
Rabbit   1280   ADVGIAIGTGTDVAIEAADVVLIRNDLLDVVASIHLSKRTVRRIRVNLVLALIYNLVGIP   1339

Human    1338   IAAGVFMPIGIVLQPWMGSAAMAASSVSVVLSSLQLKCYKKPDLERYEAQAHGHMKPLTA   1397
                IAAGVFMPIGIVLQPWMGSAAMAASSVSVVLSSLQLKCYKKPD+ERYEAQAHG MKPLTA
Rabbit   1340   IAAGVFMPIGIVLQPWMGSAAMAASSVSVVLSSLQLKCYKKPDVERYEAQAHGRMKPLTA   1399

Human    1398   SQVSVHIGMDDRWR-DSPRATPWDQVSYVSQVSLSSLTSDKPSRHSAAADDDGDKWSLLL   1456
                SQVSVH+GMDDR R DSPRAT WDQVSYVSQVSLSSLTSD+ SRHS A DD GDKWSLLL
Rabbit   1400   SQVSVHVGMDDRRRGDSPRATSWDQVSYVSQVSLSSLTSDRLSRHSPAVDDAGDKWSLLL   1459

Human    1457   NGRDEEQYI  1465
                N RDEEQYI
Rabbit   1460   NDRDEEQYI  1468
```

Fig. S4 Alignment of amino acid sequences encoding human and rabbit ATP7B protein

```
idetities  74/78 (94.87%)

Human     LLGGWYFYVQAYKSLRHRSANMDVLIVLATSIAYVYSLVILVVAVAEKAERSPVTFFDTPPMLFVFIALGRWLEHLAK
           LGGWYFYVQAYKSLRHRSANMDVLIVLATSIAYVYSL+ILVVAVAE+AERSPVTFFDTPPMLFVFI+LGRWLEH+AK
Rabbit    FLGGWYFYVQAYKSLRHRSANMDVLIVLATSIAYVYSLIILVVAVAEQAERSPVTFFDTPPMLFVFISLGRWLEHVAK
```

Fig. S5 Amino acid sequences on exon 8 of human and rabbit *ATP7B* gene

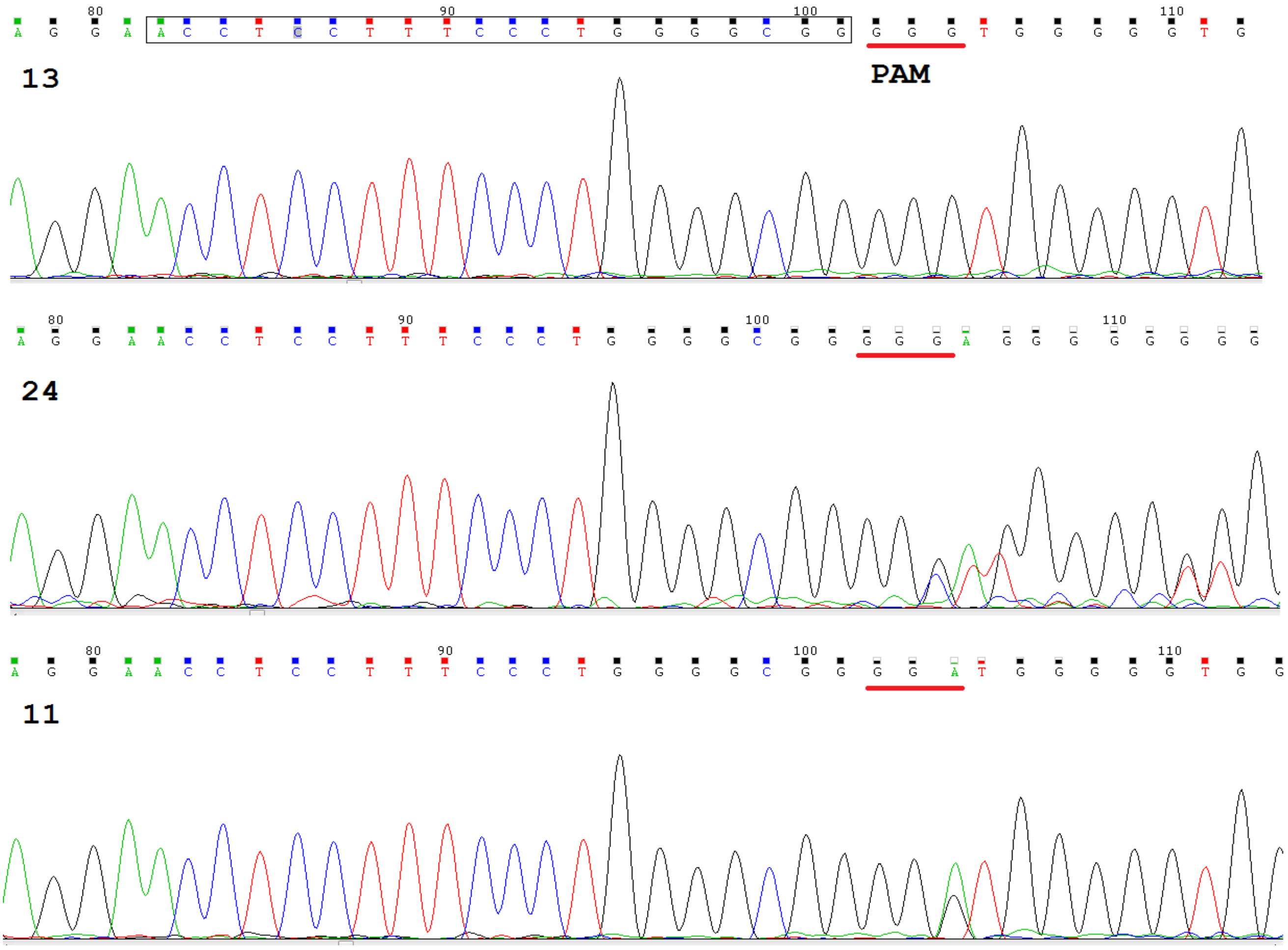

Fig. S6 The DNA sequence of fragments of potential sgRNA1F offarget site #1(Table S2)

The offtarget site was marked by black frame, and the PAM was marked by red line. 13 was the rabbit without offtarget, 24 and 11 was the rabbit with different mutations.

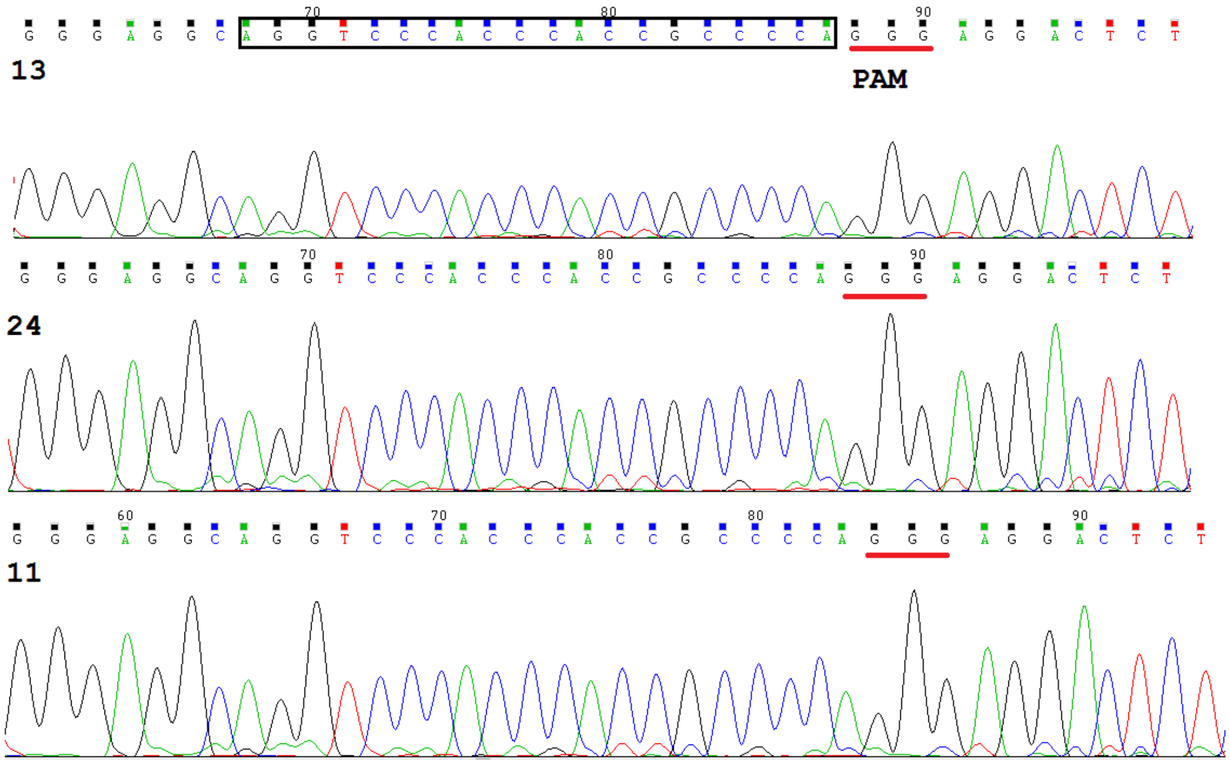

Fig. S7 The DNA sequence of fragments of potential sgRNA2R offarget site #1(Table S2)

The offtarget site was marked by black frame, and the PAM was marked by red line. 13

24 and 11was the rabbit without offtarget.

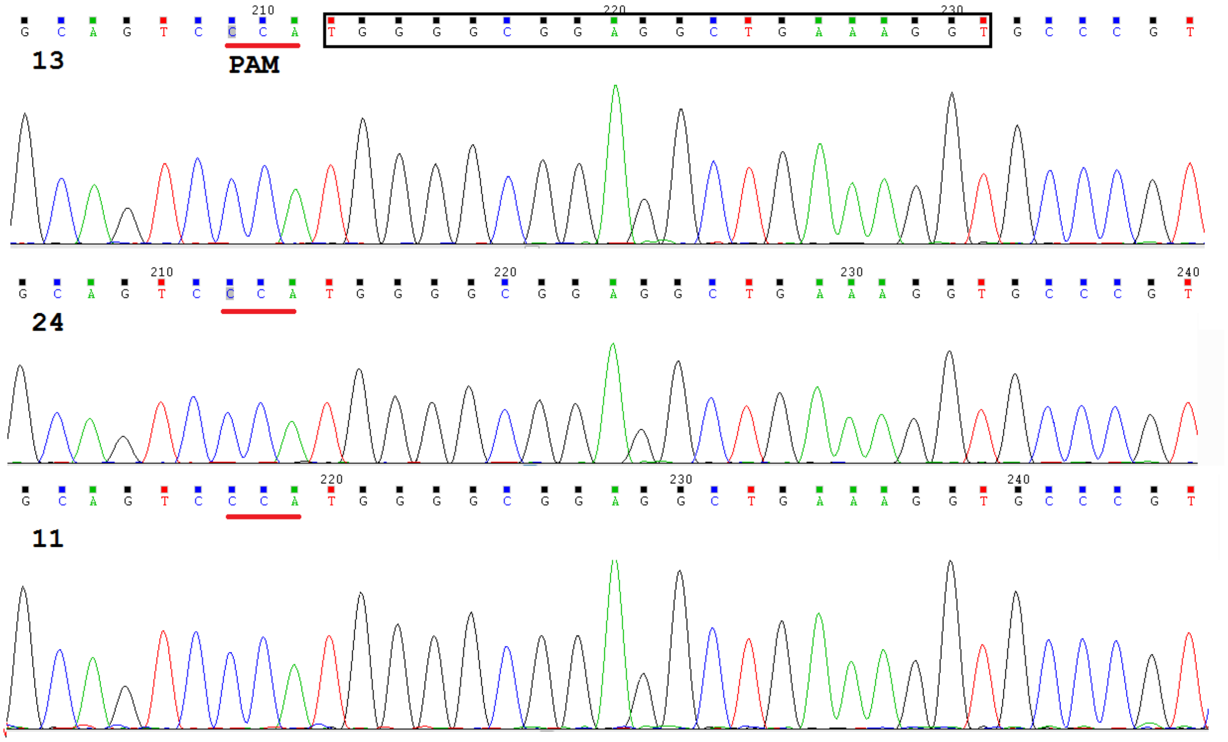

Fig. S8 The DNA sequence of fragments of potential sgRNA2R offarget site #2(Table S2)

The offtarget site was marked by black frame, and the PAM was marked by red line. 13

24 and 11was the rabbit without offtarget.

Full -length gel in the article

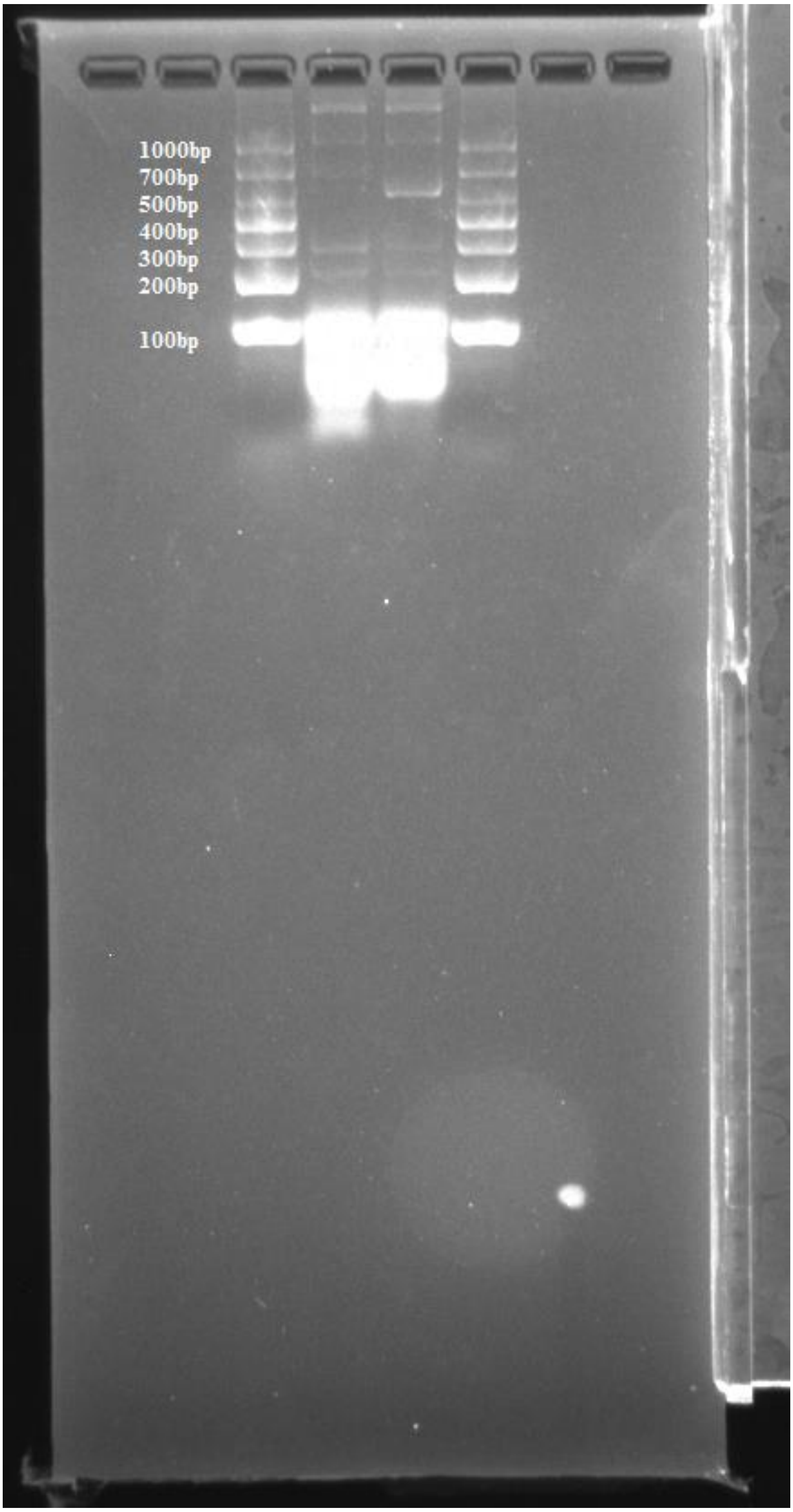

Full -length gel of Figure 2

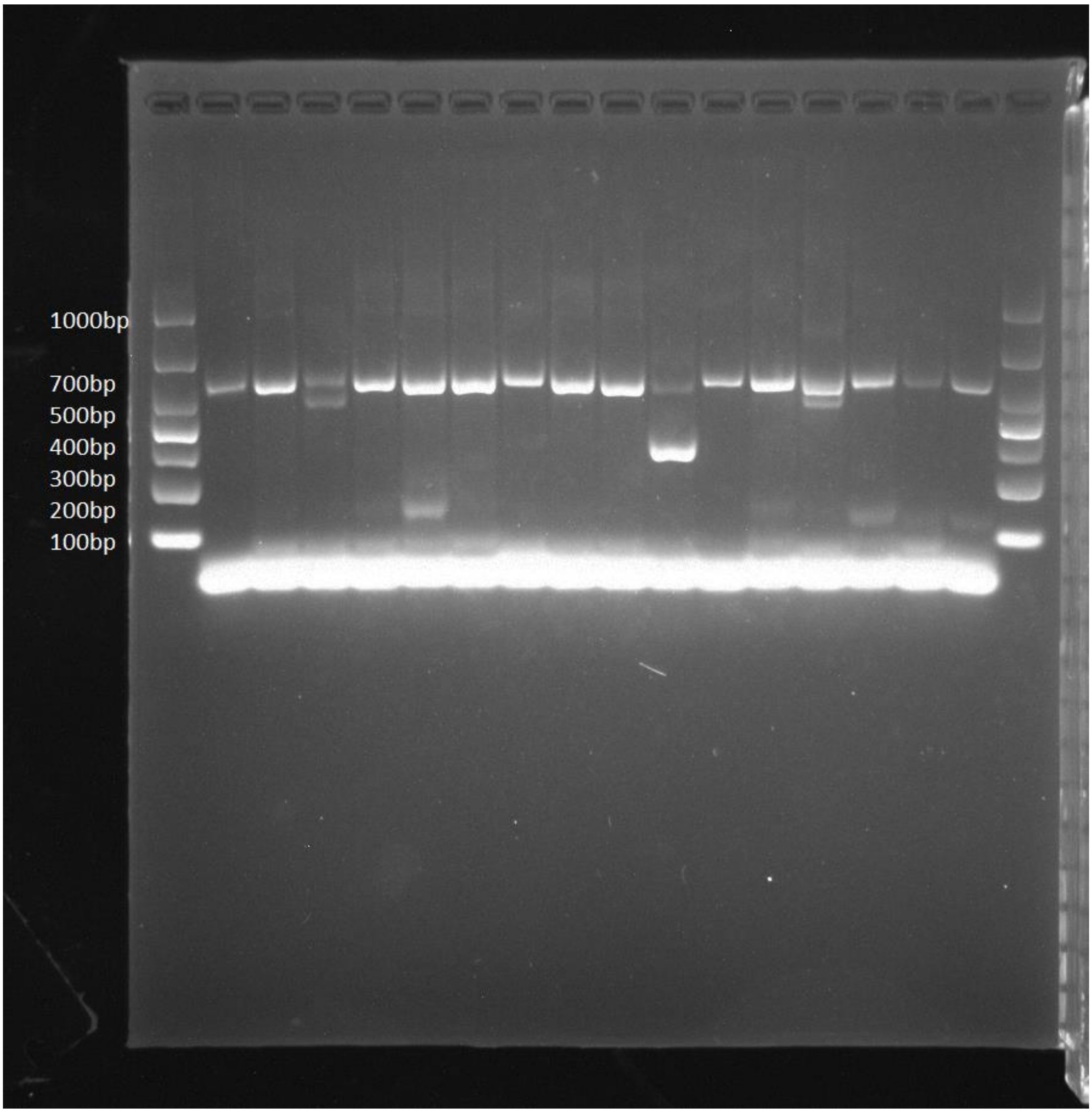

Full -length gel of Figure 3A up

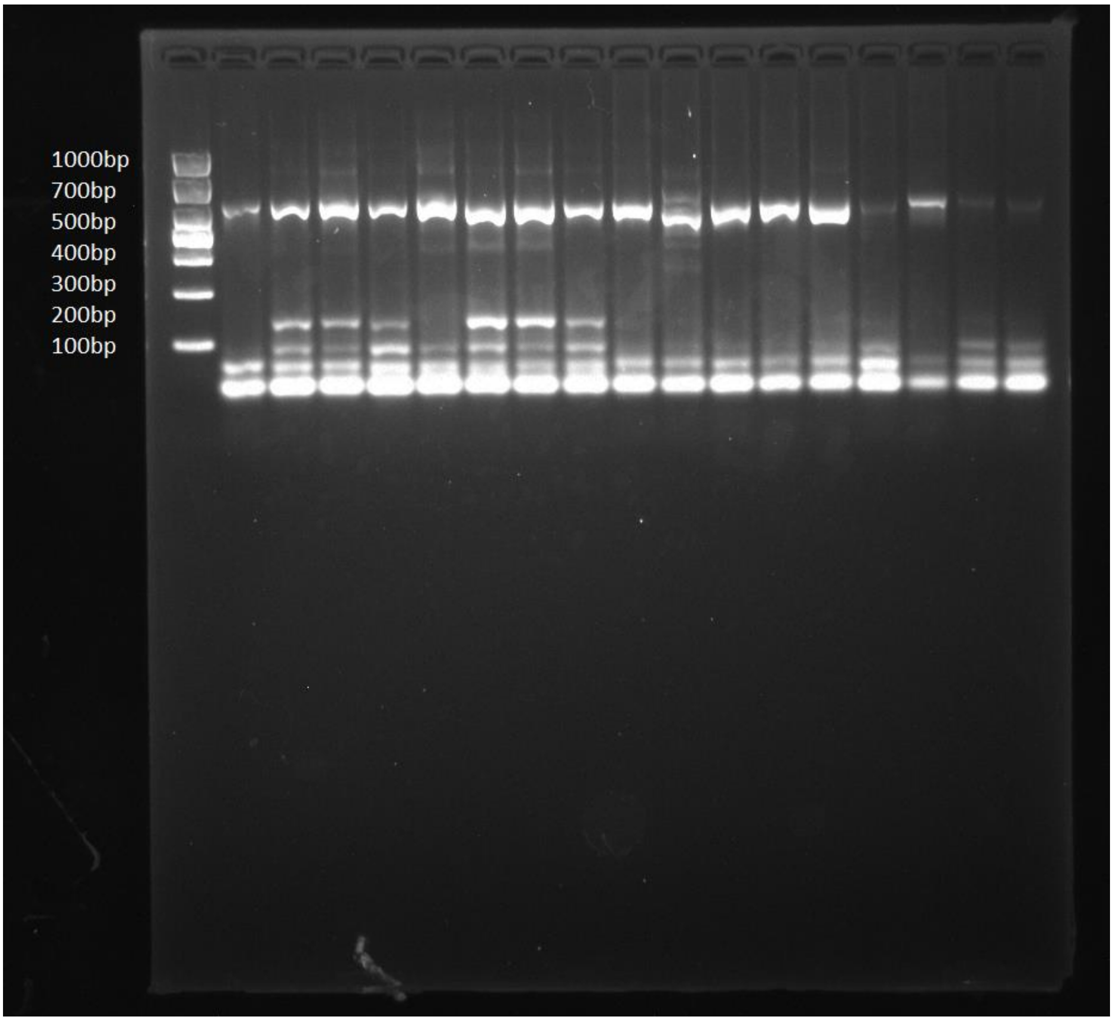

Full -length gel of Figure 3A down

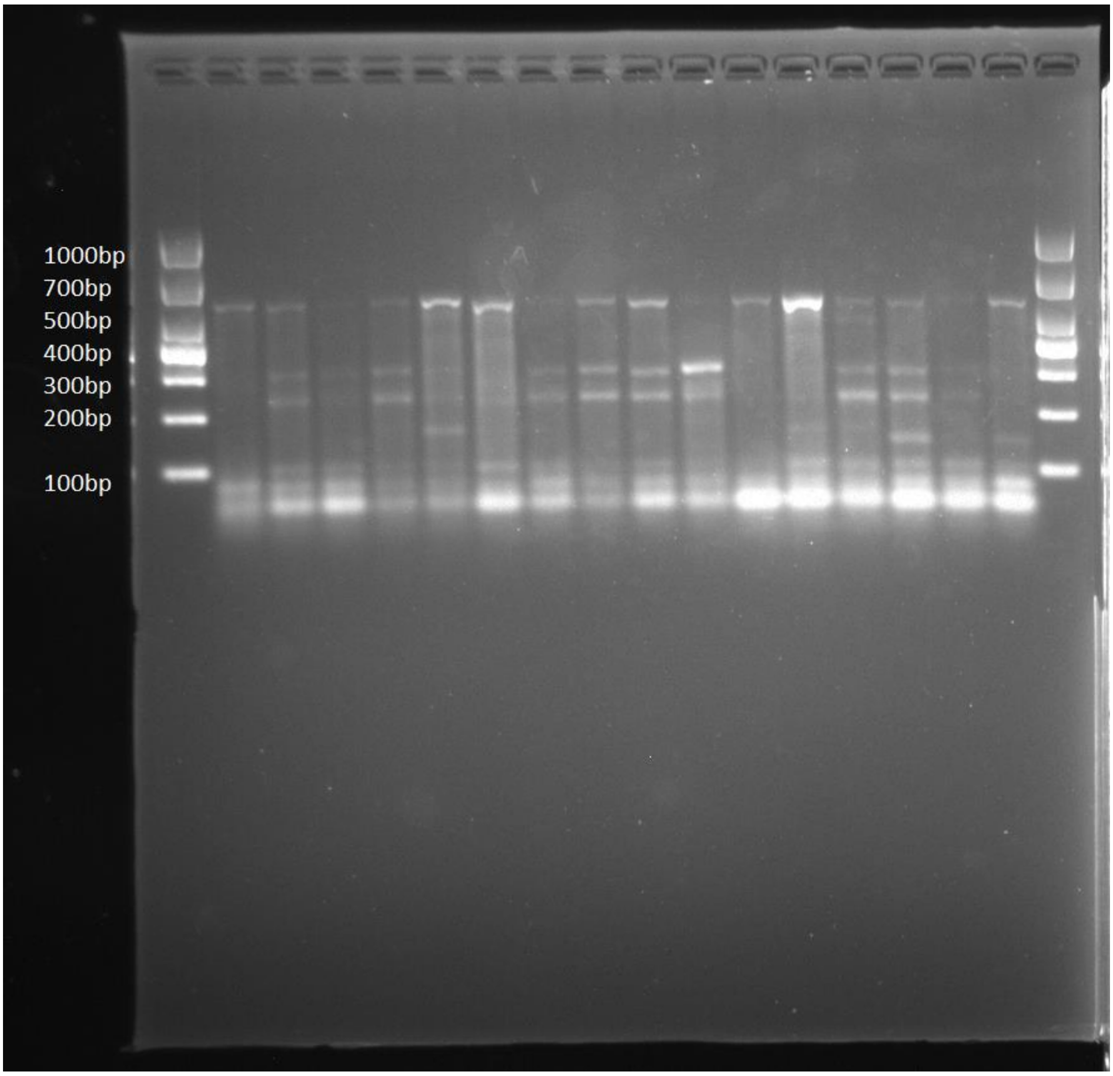

Full -length gel of Figure 3B up

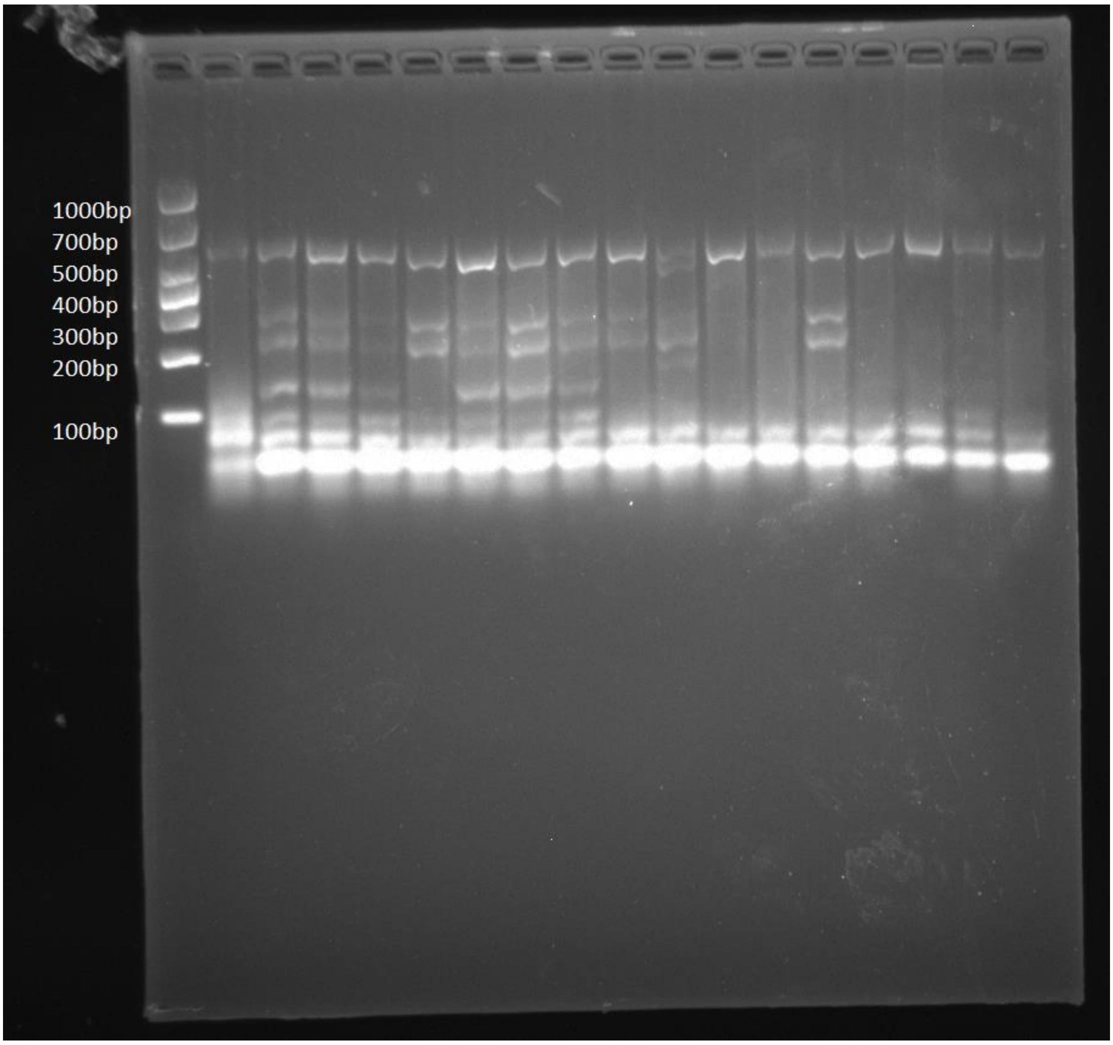

Full -length gel of Figure 3B down

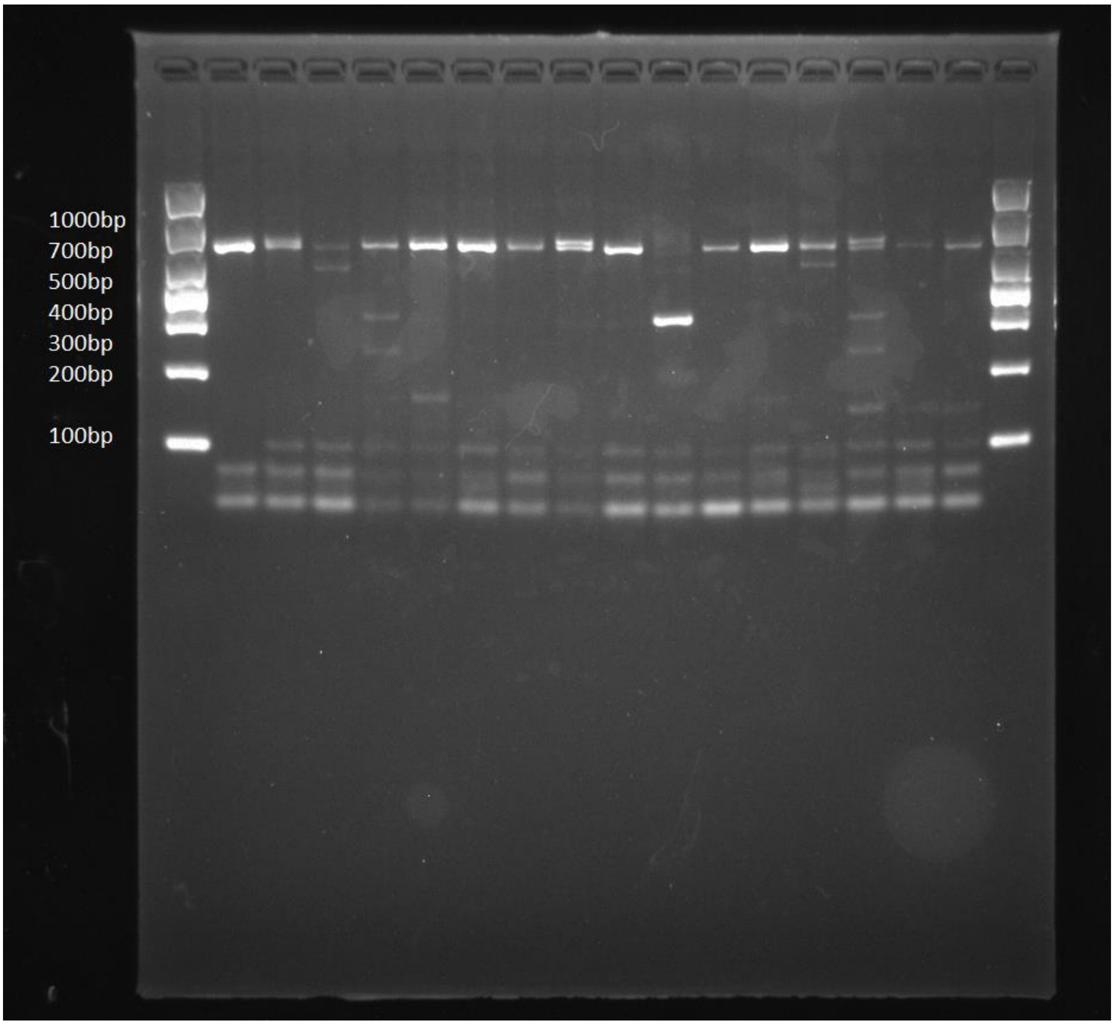

Full -length gel of Figure 3C up

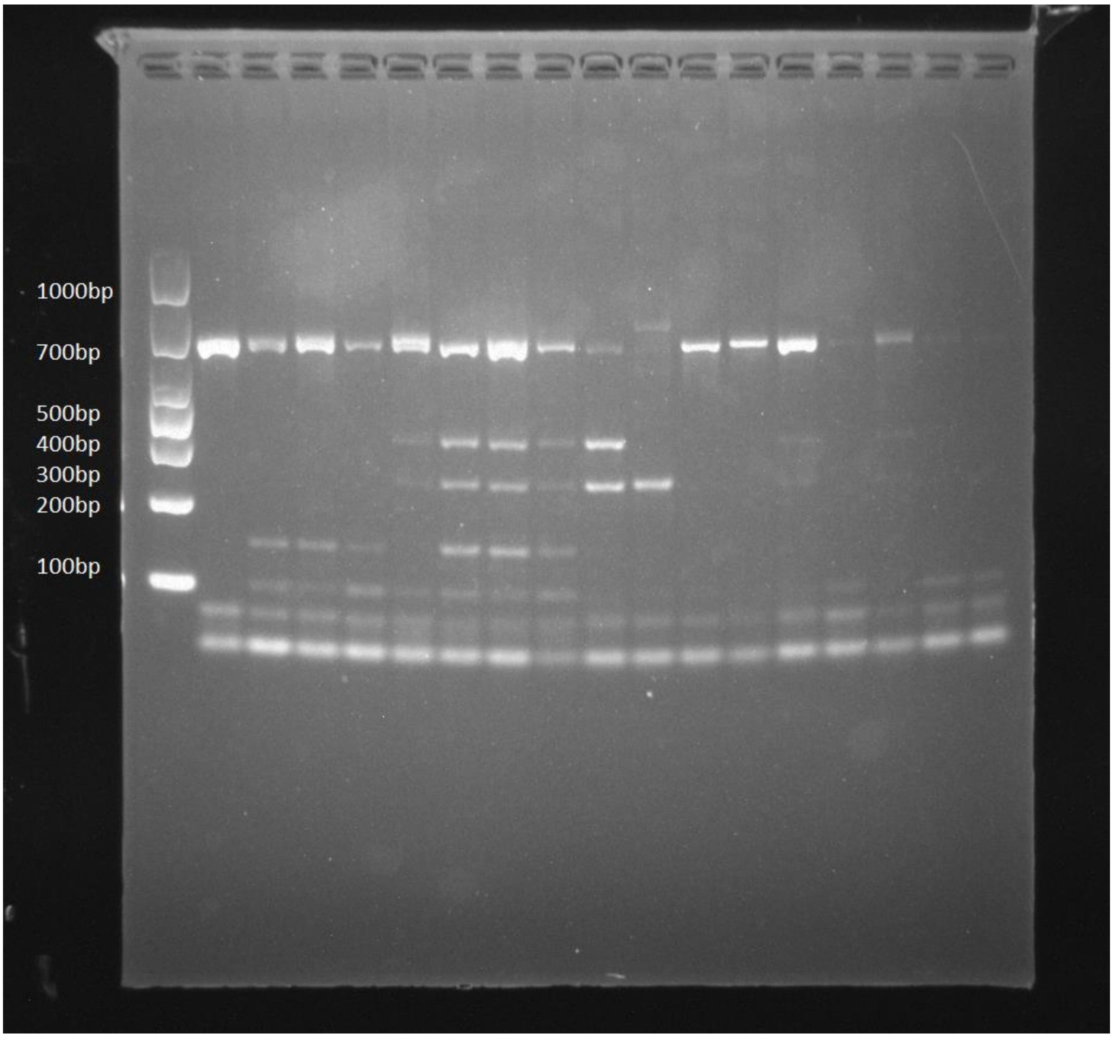

Full -length gel of Figure 3C down
